# Allelic reprogramming of chromatin states in human early embryos

**DOI:** 10.1093/nsr/nwad328

**Published:** 2024-01-02

**Authors:** Shenli Yuan, Lei Gao, Wenrong Tao, Jianhong Zhan, Gang Lu, Jingye Zhang, Chuanxin Zhang, Lizhi Yi, Zhenbo Liu, Zhenzhen Hou, Min Dai, Han Zhao, Zi-Jiang Chen, Jiang Liu, Keliang Wu

**Affiliations:** Center for Reproductive Medicine, Shandong University, Jinan 250012, China; CAS Key Laboratory of Genome Sciences and Information, Collaborative Innovation Center of Genetics and Development, Beijing Institute of Genomics, and China National Center for Bioinformation, Chinese Academy of Sciences, Beijing 100101, China; University of Chinese Academy of Sciences, Beijing 100049, China; CUHK-SDU Joint Laboratory on Reproductive Genetics, School of Biomedical Sciences, the Chinese University of Hong Kong, Hong Kong, China; CAS Key Laboratory of Genome Sciences and Information, Collaborative Innovation Center of Genetics and Development, Beijing Institute of Genomics, and China National Center for Bioinformation, Chinese Academy of Sciences, Beijing 100101, China; Center for Reproductive Medicine, Shandong University, Jinan 250012, China; Key Laboratory of Reproductive Endocrinology of the Ministry of Education, Shandong University, Jinan 250012, China; CAS Key Laboratory of Genome Sciences and Information, Collaborative Innovation Center of Genetics and Development, Beijing Institute of Genomics, and China National Center for Bioinformation, Chinese Academy of Sciences, Beijing 100101, China; University of Chinese Academy of Sciences, Beijing 100049, China; CUHK-SDU Joint Laboratory on Reproductive Genetics, School of Biomedical Sciences, the Chinese University of Hong Kong, Hong Kong, China; Center for Reproductive Medicine, Shandong University, Jinan 250012, China; Key Laboratory of Reproductive Endocrinology of the Ministry of Education, Shandong University, Jinan 250012, China; Center for Reproductive Medicine, Shandong University, Jinan 250012, China; Key Laboratory of Reproductive Endocrinology of the Ministry of Education, Shandong University, Jinan 250012, China; CAS Key Laboratory of Genome Sciences and Information, Collaborative Innovation Center of Genetics and Development, Beijing Institute of Genomics, and China National Center for Bioinformation, Chinese Academy of Sciences, Beijing 100101, China; CAS Key Laboratory of Genome Sciences and Information, Collaborative Innovation Center of Genetics and Development, Beijing Institute of Genomics, and China National Center for Bioinformation, Chinese Academy of Sciences, Beijing 100101, China; Center for Reproductive Medicine, Shandong University, Jinan 250012, China; Key Laboratory of Reproductive Endocrinology of the Ministry of Education, Shandong University, Jinan 250012, China; University of Chinese Academy of Sciences, Beijing 100049, China; Center for Reproductive Medicine, Shandong University, Jinan 250012, China; Key Laboratory of Reproductive Endocrinology of the Ministry of Education, Shandong University, Jinan 250012, China; Center for Reproductive Medicine, Shandong University, Jinan 250012, China; Key Laboratory of Reproductive Endocrinology of the Ministry of Education, Shandong University, Jinan 250012, China; Center for Reproductive Medicine, Ren Ji Hospital, School of Medicine, Shanghai Jiao Tong University, Shanghai 200135, China; CAS Key Laboratory of Genome Sciences and Information, Collaborative Innovation Center of Genetics and Development, Beijing Institute of Genomics, and China National Center for Bioinformation, Chinese Academy of Sciences, Beijing 100101, China; University of Chinese Academy of Sciences, Beijing 100049, China; CAS Center for Excellence in Animal Evolution and Genetics, Chinese Academy of Sciences, Kunming 650223, China; Center for Reproductive Medicine, Shandong University, Jinan 250012, China; Key Laboratory of Reproductive Endocrinology of the Ministry of Education, Shandong University, Jinan 250012, China

**Keywords:** human embryo, epigenetics reprogramming, DNA methylation, H3K27me3, chromatin accessibility

## Abstract

The reprogramming of parental epigenomes in human early embryos remains elusive. To what extent the characteristics of parental epigenomes are conserved between humans and mice is currently unknown. Here, we mapped parental haploid epigenomes using human parthenogenetic and androgenetic embryos. Human embryos have a larger portion of genome with parentally specific epigenetic states than mouse embryos. The allelic patterns of epigenetic states for orthologous regions are not conserved between humans and mice. Nevertheless, it is conserved that maternal DNA methylation and paternal H3K27me3 are associated with the repression of two alleles in humans and mice. In addition, for DNA-methylation-dependent imprinting, we report 19 novel imprinted genes and their associated germline differentially methylated regions. Unlike in mice, H3K27me3-dependent imprinting is not observed in human early embryos. Collectively, allele-specific epigenomic reprogramming is different in humans and mice.

## INTRODUCTION

Under natural conditions, complete mammalian parthenogenetic (PG) or androgenetic (AG) embryos cannot properly develop into an organism. Individuals with mosaic uniparental diploidy are reportedly associated with imprinting syndromes. This implies that maternal and paternal genomes carry distinct information necessary for regulating embryonic development. It has been confirmed that gene imprinting, an epigenetic mechanism explaining the parent-of-origin expression, impedes parthenogenesis or androgenesis [1,2]. Disruptions in DNA-methylation-dependent imprinting in mammals have been linked to various physiological diseases, including mental and metabolic disorders [3]. Single nucleotide polymorphisms (SNPs) have been extensively employed to distinguish paternal and maternal genomes in many species, including mice [4–7]. However, due to their complicated genetic background, informative SNPs can only track a limited proportion of human genomes [8]. Consequently, our knowledge about the patterns and roles of allele-specific epigenetic information, such as DNA methylation, histone modifications and chromatin accessibility, is limited in terms of human development.

Previous studies have indicated that PG and AG genomes can mimic maternal and paternal genomes during mouse early embryogenesis, respectively [9–12]. Human PG and AG embryos have been established in the last decade, and are useful models to investigate the differences between maternal and paternal genomes [13,14]. It was recently reported that gene expression patterns between PG and AG embryos at human early developmental stages are different [15,16]. However, the underlying molecular mechanisms regulating the differential gene expression between human PG and AG embryos remain unknown. Gene imprinting plays a critical physiological role in human development [17,18]. The dysregulation of imprinted gene expression and function can lead to many human disorders [17,19]. It is well known that allelic expression (also called allele-specific expression) of imprinted genes is regulated by parentally specific DNA methylation in imprinting control regions (ICRs) [19,20], which are classified into germline differentially methylated regions (gDMRs) and somatic DMRs [5]. In order to regulate the allele-specific expression of imprinted genes in somatic cells, gDMRs play an important role in orchestrating the establishment of other allele-specific epigenetic modifications, including somatic DMRs, within the imprinted genomic domain throughout the course of development [17]. Despite hundreds of imprinted genes and allelic DMRs having been identified in humans [18,21–26], it is still uncertain whether there are novel imprinted genes and gDMRs. Moreover, our understanding of the intricate relationship between allelic DMRs and many imprinted genes, as well as the mechanisms through which allelic DMRs modulate the expression of specific imprinted genes, remains to be further explored. Furthermore, maternal H3K27me3-dependent imprinting has been documented in mice [12]. Nevertheless, the existence of maternal H3K27me3-dependent imprinting in humans remains uncertain.

Genome-wide DNA demethylation takes place during early embryogenesis in both humans and mice [5,27–30], and then gradually re-establishes DNA methylation after implantation [31,32]. Moreover, broad H3K4me3 and H3K27ac domains are widely distributed in human and mouse early embryos before zygotic genome activation, and subsequently transform into narrow (typical) peaks [33–37]. This transition from broad domains into typical peaks is critical for normal zygotic genome activation (ZGA) in human and mouse embryos [35,37]. Utilizing DNase I hypersensitive site (DHS) sequencing, two research groups have revealed that the chromatin landscape of the entire genome displays relatively low accessibility at the four-cell and earlier stages in humans and mice, followed by a rapid and substantial increase in chromatin accessibility at eight-cell and later stages [38,39]. Although epigenetic reprogramming during early embryo development is largely conserved between humans and mice, some aspects manifest species-specific features. For instance, the distal H3K27me3 signal in the maternal genome is inherited in mouse early embryos [40], but the H3K27me3 signal is completely depleted in human eight-cell embryos [36]. OCT4 plays an important role in human ZGA but not in mice [38]. Recently, the allelic epigenomes of mouse early embryos have been extensively investigated. To what extent the similarity or difference between parental genomes in early embryos are conserved between humans and mice is still unknown.

## RESULTS

### Overall epigenetic patterns of the human paternal and maternal genome

**Figure 1.**
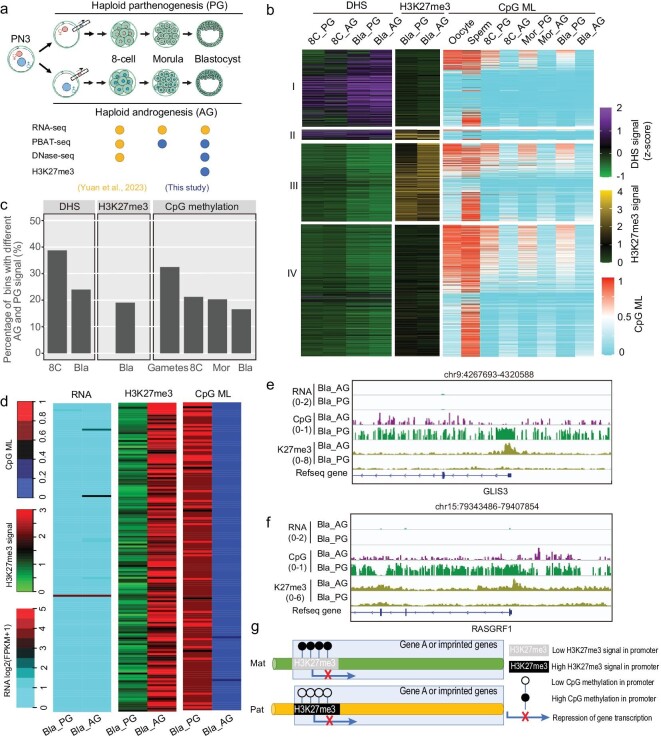
Overall epigenetic patterns in human PG and AG embryos. (a) Schematic of the generation of human haploid parthenogenetic (PG) and androgenetic (AG) embryos used for RNA-seq, post-bisulfite adaptor tagging (PBAT) sequencing, DNase l hypersensitive sites sequencing (DNase-seq) and H3K27me3 ultra-low-input micrococcal nuclease-based native ChIP-seq (ULI-NChIP-seq). The blue dots mean that experimental data are from this study, while the yellow dots mean the data are from Yuan *et al.*, 2023 [16]. For DNA methylation, four replicates of AG or PG (AG/PG) morulae were used: AG/PG_R1 (*n* = 1), AG/PG_R2 (*n* = 1), AG/PG_R3 (*n* = 2), AG/PG_R4 (*n* = 2); two replicates of AG or PG blastocysts were used: AG/PG_R1 (*n* = 1), AG/PG_R2 (*n* = 1). For DNase-seq, two replicates of AG or PG blastocysts were used: AG/PG_R1 (*n* = 1), AG/PG_R2 (*n* = 1). For ULI-NChIP-seq of H3K27me3, two replicates of AG or PG blastocysts were used: AG/PG_R1 (*n* = 2), AG/PG_R2 (*n* = 2). ‘*n*’ represents the number of embryos used in each replicate. (b) A heat map showing the DHS signal, H3K27me3 signal and CpG methylation levels (CpG ML) of genomic regions in PG and AG haploid embryos. The genome is divided into four groups of regions according to the DHS and H3K27me3 signal. Regions in group I only show a DHS signal but no H3K27me3 signal in any embryos. Regions in group II show both DHS and H3K27me3 signals in embryos. Regions in group III only show the H3K27me3 signal but no DHS signal in any embryos. Regions in group IV show neither a H3K27me3 signal nor DHS signal in any embryos. 8C represents an eight-cell embryo; Bla represents blastocyst; Mor represents morula. (c) Bar plot showing the percentage of genomic regions (bins) with differential DHS signal (left), H3K27me3 signal (middle) and DNA methylation (right) between AG (paternal) and PG (maternal) embryos. The percentage of differential DHS at eight-cell stage is calculated by the ratio of *m*/*n*, where *m* indicates the number of regions marked by the parentally specific DHSs in the eight-cell embryos, and *n* indicates the number of regions marked by the DHSs in either PG or AG eight-cell embryos. Similar methods were applied to calculate the percentages of regions with differential H3K27me3 signal or DNA methylation. (d) A heat map showing gene expression, DNA methylation and H3K27me3 signal in the gene promoters with both PG-specific DNA methylation and AG-specific H3K27me3 at blastocyst stage. (e–f) Genome browser view of DNA methylation and H3K27me3 signal in two imprinted genes, *GLIS3* (e) and *RASGRF1* (f). (g) A schematic model showing the relationship between parentally specific epigenetic status and gene expression. Paternally (Pat, also as AG) specific H3K27me3 and maternally (Mat, also as PG) specific DNA methylation could be associated with repression of the alleles.

In order to examine the differences between paternal and maternal epigenomes in humans, we generated and collected PG and AG embryos at the morula and blastocyst stages (see Methods) (Fig. 1a). The embryos with high morphological qualities were collected to map the chromatin accessibility landscapes, DNA methylomes, H3K27me3 modification patterns and transcriptomes (Fig. 1a and Table S1). We also integrated the published data [16], which include AG and PG transcriptomes, along with the DHSs and DNA methylation in AG and PG embryos at eight-cell stage (Fig. 1a). Initially, we conducted multiple analyses to validate our data. As we know, chromatin accessibility and H3K27me3 play crucial roles in the regulation of gene expression. As expected, genes with promoter DHSs exhibited elevated expression levels compared to those lacking promoter DHS (Fig. S1a). Consistent with the fact that H3K27me3 is a repressive marker, the genes with an H3K27me3 signals in promoters show lower expression than those without an H3K27me3 signals in promoters in PG and AG embryos (Fig. S1b). We also evaluated the relationship between different epigenetic states. We classified the human genome into four groups according to DHS and H3K27me3 signals (Fig. 1b). Consistent with previous reports, the DHSs are, overall, anticorrelated with repressive epigenetic marks including DNA methylation and H3K27me3 in both human AG embryos and PG embryos (Fig. 1b and Fig. S1c). A small number of genomic regions are marked with both DHSs and H3K27me3 (Fig. 1b, group II). We found that genes whose promoters had both DHSs and H3K27me3 exhibited lower expression levels than those with only DHSs (Supplementary Fig. S1d). In addition, H3K27me3 signals are overall reversely correlated with DNA methylation levels in both AG and PG blastocysts (Fig. 1b, group II and III). Taken together, the qualities of epigenomes data in human PG and AG embryos are high.

Next, we evaluated the epigenetic differences between maternal and paternal genomes in human early embryos (Fig. 1c). Of DHSs, 39% and 24% show differential signals between parental genomes at the eight-cell and blastocyst stages, respectively (Fig. 1c). Of H3K27me3-marked regions, 19% show parentally specific signals at the blastocyst stage (Fig. 1c). Of genomic regions, ∼20% are parentally specifically methylated in human early embryos (Fig. 1c). Collectively, our data show that a significant proportion of genomic regions present distinct epigenomic patterns between maternal and paternal genomes.

Moreover, we explored the relationship of allelic states among different kinds of epigenetic modifications. In regions exhibiting parentally specific DHSs, our data indicate that those with AG-specific DHSs are generally DNA hypomethylated in the AG embryo, while being relatively hypermethylated in the PG embryo (Fig. S1e, f). However, an exception is observed for the PG-specific DHSs at the eight-cell stage, which are DNA unmethylated in both PG and AG embryos (Fig. S1e, f). Intriguingly, the regions with AG-specific H3K27me3 signals are DNA hypomethylated in AG embryos, but DNA hypermethylated in PG embryos (Fig. 1c and Fig. S1g, h). These regions cover the promoters of 159 genes, and most of the genes show low expression in AG and PG blastocysts (Fig. 1d and Fig. S2a, b). Gene ontology (GO) analysis shows that these genes are enriched in neuron activity (Table S2). In addition, ∼20% (31 in 159) of the genes are imprinted genes (Fig. 1e, f and Table S2). Moreover, 61 of 159 genes are associated with reported allelic DMRs (Table S2). These results indicate that maternal DNA methylation and paternal H3K27me3 may coordinate to repress the expression of developmental or imprinted genes at early embryo stages (Fig. 1g). Furthermore, for the 159 genes, our data show that DNA hypermethylation of maternal alleles is inherited from oocytes (Fig. 1b and Fig. S2c). ∼78% of promoters are lowly methylated or unmethylated in sperm, whereas the majority of the remaining promoters are highly methylated in sperm, which will undergo DNA demethylation in the early embryonic stages (Fig. S2c). Taken together, allelic DNA methylation and allelic H3K27me3 cooperate to be associated with the repression of gene expression in human early embryos.

We are interested in whether the cooperation of allelic DNA methylation and H3K27me3 to repress gene transcription in early embryos is conserved between humans and mice. We analyzed the data of DNA methylation [5] and H3K27me3 landscapes [40] in mouse embryos at the blastocyst stage. We observed that the promoters of *Olfr76* and *Gas2* with paternally specific H3K27me3 signals are DNA hypermethylated in the maternal genome (Fig. S2d, e), while the other regions of these two genes are both DNA hypermethylated in maternal and paternal genomes. *Olfr76* and *Gas2* are both developmental genes, involved in olfactory receptor activity and primordial ovarian follicle growth, respectively. RNA-seq data show that both of these two genes are not expressed in mouse inner cell mass (ICM) (Fig. S2d, e). Taken together, it is a conserved mechanism that the repression of two parental alleles of developmental genes is associated with allelic DNA methylation and H3K27me3, respectively.

### Allelic chromatin accessibility in human early embryos

**Figure 2.**
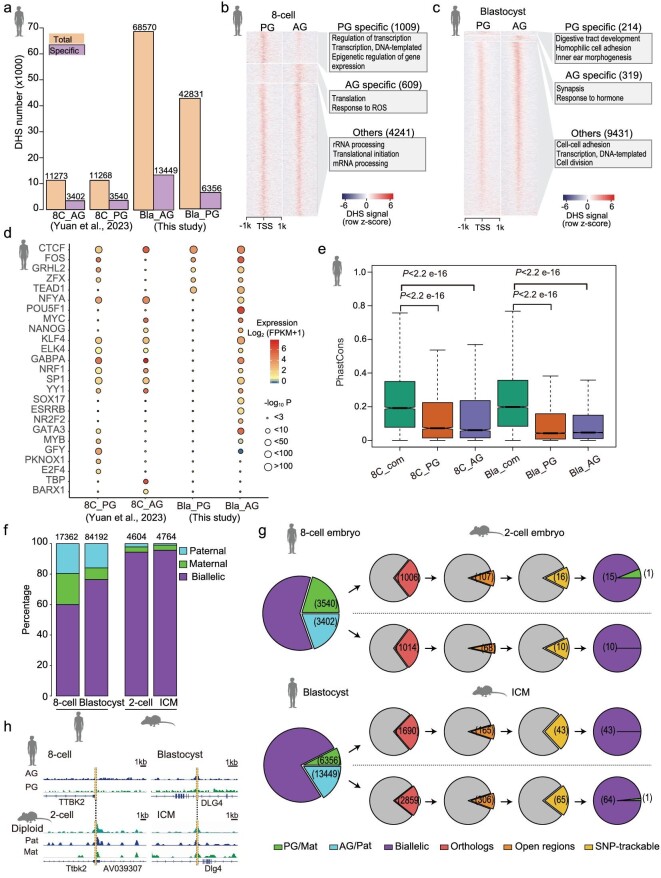
Characteristics of allelic chromatin accessibility in human embryos. (a) Bar plots showing the number of total DHSs and parentally specific DHSs in human PG and AG haploid embryos. (b–c) Heat maps showing the DHS signal in the protein-coding gene promoters with parentally specific DHS at the eight-cell (b) and blastocyst (c) stages. The numbers of promoters with parentally specific DHS and GO items of the genes are indicated on the right of the heat map. (d) Enrichment of transcription factor (TF) binding motifs within parentally specific DHSs at the eight-cell and blastocyst stages. The sizes of circles represent the *P-*values of enrichment. The colors in the circles represent the expression levels of TFs in the corresponding PG or AG embryos. (e) Conservation analysis of bi-allelic (com) and allele-specific DHSs. Wilcoxon rank sum test was used. The DHSs with higher phastCons values are more conserved. (f) A bar plot comparing the percentages of maternally (PG) specific, paternally (AG) specific and bi-allelic DHSs between human and mouse embryos. (g) Pie charts showing the distribution of the orthologous regions of human parentally specific DHS in mouse embryos. For example, for 3540 PG-specific DHSs in the human eight-cell embryo, 1006 regions have orthologous regions in the mouse genome. Of the 1006 regions, 107 show an open chromatin state in the mouse embryo, in which only 16 regions can be tracked by parental SNP information. For the 16 regions, only 1 region shows a maternally specific signal. (h) Genome browser view of chromatin accessibility in orthologous regions (shadow regions) between humans and mice.

Previous studies have shown that the number of DHSs before the human ZGA stage is very limited, and a large number of DHSs are gradually established after the eight-cell stage [38]. Consistently, there are more DHS regions at the blastocyst stage than the eight-cell stage for both PG and AG embryos (Fig. 2a). Here, we identified allele-specific DHSs (see Methods). Different to the comparable parentally specific DHSs at eight-cell stage [16], 13 449 AG-specific DHSs and 6356 PG-specific DHSs are detected at the blastocyst stage (Fig. 2a). A substantial number of allele-specific DHSs reside in intergenic regions, yet a significant fraction can also be found within promoters (Fig. S3a). To investigate whether these intergenic DHSs can generate spurious transcripts or are potentially distal enhancers of genes, we explored the expression of AG- or PG-specific intergenic DHSs in human AG and PG blastocysts. 11.8% AG-specific DHSs and 13.9% PG-specific DHSs are expressed (reads per kilo base per million mapped reads, RPKM >= 1) in AG and PG blastocysts respectively (Fig. S3b, c). This finding suggests that a minority of these DHSs may generate spurious transcripts. Furthermore, 27.1% of AG-specific intergenic DHSs and 17.8% of PG-specific intergenic DHSs are associated with H3K27ac peaks in human blastocysts (Fig. S3d). This result implies that a fraction of DHSs in intergenic regions may function as potential distal enhancers for genes. Compared to the DHSs in eight-cell embryos, most of the AG- or PG-specific DHSs in blastocysts are newly established during eight-cell to blastocyst transition (Fig. S3e). ∼100 AG-specific DHSs and 42 PG-specific DHSs are maintained between eight-cell and blastocyst stages in AG and PG embryos (Table S3). It is well-established that DHSs usually mark active *cis*-regulatory elements, including promoters and enhancers, which are occupied by transcription factors (TFs) to activate gene transcription. Firstly, we investigated DHS signals in the gene promoters in PG and AG embryos. The majority of promoters with DHSs exhibit comparable DHS signals between PG and AG embryos at both eight-cell and blastocyst stages (Fig. 2b, c). However, there are 1009 promoters with PG-specific signals and 609 promoters with AG-specific signals at eight-cell stage, whereas 214 and 319 promoters harbor PG- and AG-specific DHS signals at blastocyst stage, respectively (Fig. 2b, c). At blastocyst stage, GO analysis indicates that the protein-coding genes with PG-specific promoter DHSs are enriched in organ development and homophilic cell adhesion, the genes with AG-specific promoter DHSs are enriched in synapsis and hormone response, while the genes with both PG and AG DHSs are enriched in cell–cell adhesion and transcription. In addition, allelic DHS signals in promoters are associated with allelic gene expression. In blastocysts, 29 genes with AG-specific promoter DHSs show AG-specific expression (Fig. S3f), and 6 genes with PG-specific DHSs show PG-specific expression (Fig. S3g).

Next, we explored the TF binding motifs in the DHSs. Our data indicate that the binding motif of CTCF is enriched in both PG and AG DHSs (Fig. 2d). The binding motif of GRHL2, which is involved in primary neurulation and epithelial development, is specifically enriched in PG-specific DHSs at the eight-cell stage (Fig. 2d). NFYA is important to establish a chromatin accessibility landscape at ZGA stage. Its binding motif is enriched in both PG and AG DHSs at eight-cell stage, but it is more enriched in AG-specific DHSs than PG-specific DHSs at blastocyst stage (Fig. 2d). Notably, pluripotent TFs, such as OCT4 (POU5F1), KLF4, NANOG and MYC exhibit higher binding motif enrichment in AG-specific DHSs than PG-specific DHSs in blastocysts, and their expression levels are higher in AG embryos than PG embryos (Fig. 2d). This suggests that the pluripotent TFs may play broader regulatory functions in the paternal genome than maternal genome.

The sequences of *cis*-elements vary during evolution. We were curious about the conservation of these allele-specific DHSs during evolution. Our data show that those AG- or PG-specific DHSs are less conserved in DNA sequences than common DHSs, which are detected in both AG and PG embryos (Fig. 2e).

We also investigated whether allele-specific chromatin accessibility is conserved between humans and mice. Compared to humans, proportions of allele-specific chromatin accessibility are obviously lower in mice at both the two-cell and blastocyst stages (Fig. 2f). This result suggests that the chromatin accessibility of parental genomes is more divergent in humans than in mice. To answer the question of whether the allele-specific DHSs in human early embryos are also in open chromatin states in mice, we aligned the human allele-specific DHSs in the mouse genome. Only ∼10% of the orthologue regions are also open in mouse embryos. Among the open chromatin regions that can be SNP tracked between C57BL/6N and DBA/2N strains in mice, almost all of those regions show bi-allelic ATAC (assay for transposase accessible chromatin) signals in hybrid mouse embryos (Fig. 2g, h). These results suggest that the allele-specific DHS patterns of orthologous regions are not conserved between humans and mice (Fig. 2g, h).

### Allelic DNA methylation in human early embryos

**Figure 3.**
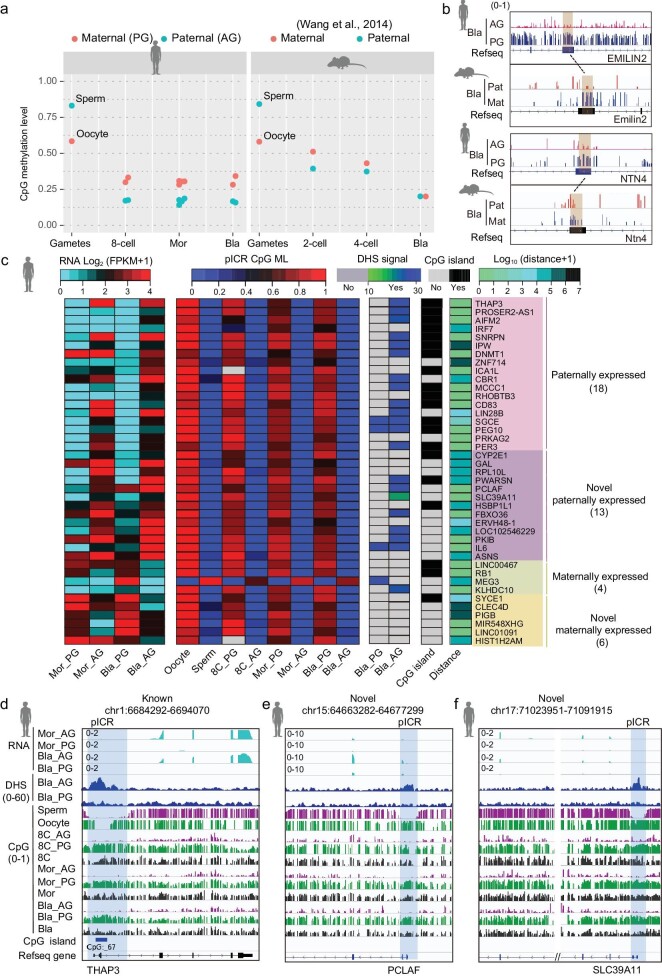
Allelic DNA methylation and imprinted genes in human embryos. (a) A plot showing the global DNA methylation levels of parental genomes in human (left) and mouse (right) gametes and early embryos. (b) Genome browser view of DNA methylation in orthologous regions (shadow regions) between humans and mice. The top orthologous regions show maternally specific DNA methylation both in humans and mice. The bottom orthologous regions show maternally specific DNA methylation in humans, but not in mice. (c) A heat map showing the RNA expression pattern of imprinted genes with AG- or PG-specific expression at the blastocyst stage and the characteristics of their putative germline ICRs (pICRs). Both known and novel imprinted genes are included. The CpG ML and DHS signal of pICRs in gametes or embryos are shown. Whether the pICRs are located in CpG islands is indicated. The distances between imprinted genes and their pICRs are also shown. The gray color in CpG ML means that the ML information is unavailable. (d–f) Genome browser view of DNA methylation, DHS signal and RNA expression pattern at imprinted genes. *THAP3* (d) is a known imprinted gene; *PCLAF* (e) and *SLC39A11* (f) are two novel imprinted genes. The blue shading indicates pICR region.

Whole-genome demethylation is a hallmark of mammalian early embryogenesis. Consistent with DNA methylome dynamics in human early bi-parental embryos [27–30], both PG and AG genomes present DNA demethylation dynamics (Fig. 3a and Fig. S4a, b). Moreover, DNA methylation levels of AG embryos are lower than those of PG embryos at several stages (Fig. 3a), which is consistent with previous results using SNPs to track paternal and maternal DNA methylation in bi-parental embryos (Fig. S4c) [29]. This indicates that the epigenetic patterns of AG and PG embryos can represent the paternal and maternal epigenetic patterns of bi-parental embryos, respectively. However, the global methylation level between maternal and paternal methylomes in mice is similar (Fig. 3a).

To investigate the differences between parental methylomes, we identified DMRs between PG and AG embryos. More DMRs with hypermethylation levels were observed in PG embryos. Most DMRs with PG hypermethylation were located in genic regions, whereas DMRs with hypermethylation in AG were distributed in intergenic regions (Fig. S4d). DNA methylation plays an important role in repressing gene expression. We further investigated the effect of differential DNA methylation patterns between PG and AG blastocysts on gene expression. Our data indicate that 37 genes whose promoters are PG specifically methylated show lower expression levels in PG blastocysts than AG blastocysts (Fig. S4e and Table S4).

For the DMRs with PG-specific DNA methylation in human blastocysts, we were curious about whether their orthologue regions in mice also manifest an allele-specific DNA methylation pattern at the blastocyst stage. The results show that most of the orthologue regions (82%, 578 of 708 regions) show bi-allelic DNA methylation or hypomethylation in mouse blastocysts (Fig. 3b and Fig. S4f). Only 16% (111/708) regions exhibit maternally specific DNA methylation in mice (Fig. 3b, and Table S5). This suggests that most of the orthologue regions do not show conserved allelic DNA methylation patterns between humans and mice.

### Imprinted genes in humans

Gene imprinting plays a critical physiological role in humans [17,18]. Allele-specific expression of imprinted genes is regulated by parentally specific DNA methylation in ICRs. In humans, 217 imprinted genes and 797 allelic DMRs have been identified [18,21–26]. Taking advantage of genome-wide allele-specific expression and DNA methylation between AG and PG embryos, we seek to identify novel imprinted genes and ICRs in the human genome (see Methods). Firstly, to find ICR regions for the imprinted genes, we performed differential DNA methylation analyses among gametes, and PG and AG embryos. We call a region a candidate germline ICR or gDMR if the region is hypomethylated in one allele and hypermethylated in the other allele at the gamete, morula and blastocyst stages (see Methods). In addition, the candidate germline ICRs should be intermediately methylated in either the placenta (placenta-specific), six-week embryo (embryo-specific) or both tissues (non-specific) in humans (see Methods). We identified 2577 gDMRs between paternal and maternal genomes. Among these DMRs, 143 regions are putative germline ICRs (pICRs) linked to 190 reported imprinted genes (see Methods) (Table S6). Out of the 143 pICRs, 81 overlap with previously reported allelic DMRs (Table S6). Most of the 143 pICRs maternally imprint with PG-specific hypermethylation (Fig. 3c, d and Table S6). In addition, many of these pICRs show AG-biased DHS signals and are located in gene promoters (Fig. 3c, d and Table S6).

Next, we attempted to identify novel imprinted genes by integrated analysis of DMRs and allelically expressed genes in PG and AG embryos. In total, we found 45 imprinted genes, including 26 well-known imprinted genes and 19 novel imprinted genes (Fig. S5a and Table S6). Most of the 26 well-known imprinted genes show consistently allele-specific expression at the blastocyst stage as reported, including 21 AG-specifically expressed genes (such as *SNRPN, CBR1, RNF141, THAP3* and *DNMT1*) and 5 PG-specifically expressed genes (such as *RB1* and *MEG3*) (Fig. S5a and Table S6). Among the 19 novel putative imprinted genes, 13 imprinted genes show AG-specific expression and 6 imprinted genes show PG-specific expression (Fig. S5a and Table S6). PCLAF is a PCNA-binding protein that acts as a regulator of DNA repair during DNA replication. Our data show that *PCLAF* is AG-specifically expressed and has an ICR in its promoter (Fig. 3e). SLC39A11 is involved in the transport of Zn^2+^ and associated with Alzheimer’s disease, and also shows AG-specific expression and has an ICR in its promoter (Fig. 3f). Our data also show that both *PCLAF* and *SLC39A11* have AG-specific promoter DHSs.

In addition, we noticed that 154 reported imprinted genes are not expressed in the human embryo before implantation. We cannot say whether these are imprinted genes or not. However, 37 reported imprinted genes show gene expression but without allele-specific expression (Table S6), indicating that those genes are not imprinted genes, at least in human early embryos. To confirm these genes are not imprinted in human early embryos, we examined the expression of these genes in previously reported human PG and AG morulae [15]. Similarly, none of the genes show allelic expression as reported (Fig. S5b).

### Allelic H3K27me3 signal in human blastocysts

**Figure 4.**
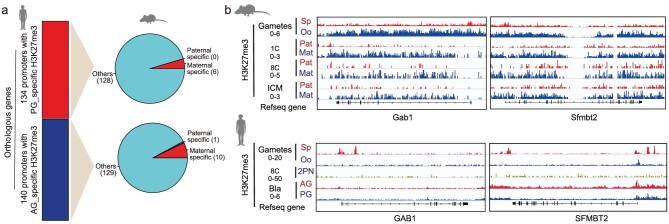
Allelic H3K27me3 patterns of orthologous promoters are not highly conserved between humans and mice. (a) Pie charts summarizing the H3K27me3 status of parental genomes in the promoters of mouse orthologous genes, which have promoters that show an AG- or PG-specific H3K27me3 signal in human blastocysts, in mouse embryos at the blastocyst stage. The H3K27me3 status of parental genomes includes maternally specific H3K27me3, paternally specific H3K27me3 and others. The numbers of orthologous genes are shown in parentheses. (b) Genome browser view of the H3K27me3 signal at *GAB1* and *SFMBT2* loci in the gametes and early embryos of mice and humans. *GAB1* and *SFMBT2* are reported to show H3K27me3-dependent imprinting in mice.

It has been reported that the H3K27me3 signal disappears at the eight-cell stage and is re-established at the later stages in human embryos [36,41]. We analyzed H3K27me3 patterns in human PG and AG blastocysts. We identified genomic regions with a differential H3K27me3 signal between PG and AG embryos. Among these regions, 76.9% (10 166/13 217) show AG-specific H3K27me3 signals, while 23.1% (3051/13 217) show PG-specific H3K27me3 signals (Fig. 1c and Fig. S1g). In contrast, only 23.9% (3342/13 997) of the regions with allelic H3K27me3 signals show a paternally specific H3K27me3 signal in mouse ICM [40]. These results indicate that the paternally specific H3K27me3 signals is predominant in humans but not in mice. We further assessed the impact of allelic H3K27me3 signals on gene expression. The majority of genes with parentally specific H3K27me3 signals in promoters are not expressed in human blastocysts; only seven genes with AG-specific H3K27me3 signals in promoters show PG-specific expression, and seven genes with PG-specific H3K27me3 signals in promoters show AG-specific expression in blastocysts (Fig. S6a, b). To answer the question of whether the orthologue genes harbor conserved allele-specific H3K27me3 signals in their promoters between humans and mice, we compared the H3K27me3 patterns in human and mouse embryos at blastocyst stage. The results show that most orthologue genes with allelic promoter H3K27me3 signals in humans do not exhibit allelic H3K27me3 signals in mice (Fig. 4a).

Recently, H3K27me3-dependent maternal imprinting has been discovered in mouse pre-implantation embryos. This is inherited from oocytes, and maintained until blastocyst stage [12]. Although H3K27me3-dependent (non-canonical) imprinting is largely lost in mouse post-implantation embryos, some non-canonical imprinted genes (such as *Gab1, Phf17*) maintain their imprinted expression in the extra-embryonic (ExE) cell lineage [12]. Mechanically, the allelic H3K27me3 to allelic DNA methylation (somatic DMR) switch maintains non-canonical imprinting in the ExE cell lineage at the period ranging from the E4.0 to E6.5 stage [4]. In mouse early embryos, 76 H3K27me3-dependent imprinted genes have maternally specific H3K27me3 regions around their gene regions [12]. However, none of the orthologous genes in humans show PG-specific H3K27me3 around their gene regions at the blastocyst stage (Fig. 4b and Fig. S6c). Besides, the regions with PG- or AG-specific H3K27me3 signals at the blastocyst do not harbor H3K27me3 signals in the eight-cell embryo. These results suggest that H3K27me3-dependent imprinting is not conserved between mice and humans.

### The allelic expression of orthologous genes is not conserved between humans and mice

**Figure 5.**
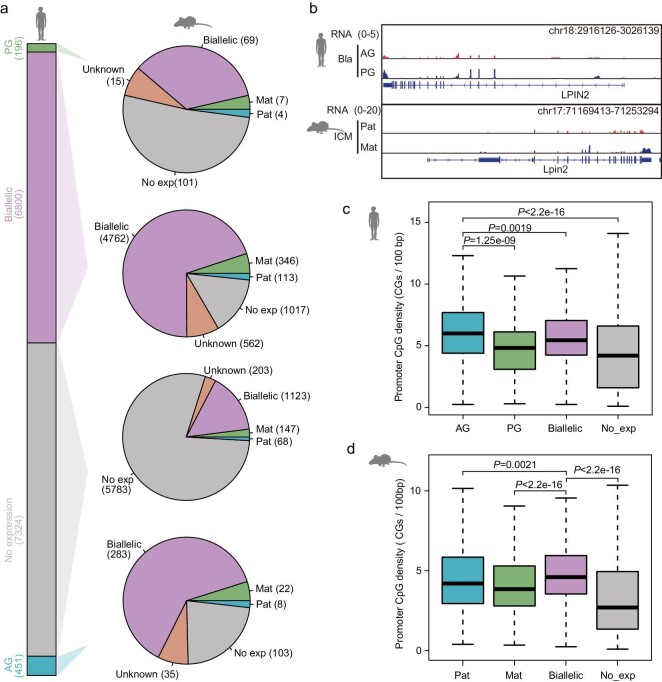
Allelic expression of orthologue genes in human and mouse embryos. (a) Pie charts summarizing the expression status of the orthologous genes in parental genomes in human and mouse embryos at the blastocyst stage. The expression status of the orthologous genes includes maternally (PG) specific expression, paternally (AG) specific expression, bi-allelic expression, no expression and unknown expression. The genes whose parental transcripts cannot be distinguished by parental SNPs are referred to as genes with unknown expression. (b) Genome browser view of the parental RNA expression of *LPIN2* in human and mouse embryos. *LPIN2* shows maternally specific expression in both humans and mice. (c–d) A boxplot comparing the promoter CpG densities of the orthologous genes with maternally specific expression, paternally specific expression, bi-allelic expression and no expression in human (c) and mouse (d) embryos at blastocyst stage. Wilcoxon rank sum test was used.

Our previous study has identified the differentially expressed genes (DEGs) between PG and AG embryos at the blastocyst stage including 568 AG-specifically expressed genes and 298 PG-specifically expressed genes [16]. The AG-specifically expressed genes are enriched in the generation of carbohydrate and energy, while PG-specifically expressed genes are enriched in cell secretion regulation and cognition. Next, we asked whether these DEGs exhibit conserved allelic expression between humans and mice. The results show that except for some DEGs that are not expressed in the mouse embryo, the majority of human DEGs are bi-allelically expressed at the blastocyst stage in mice (Fig. 5a). Only a small portion of orthologue genes share conserved allelic expression patterns between humans and mice (Fig. 5a, b). We also evaluated the features of allelically expressed genes in humans and mice. In human blastocysts, the promoters of genes with AG-specific expression show the highest CpG densities of all promoters of other types of genes, including PG-specifically expressed genes, bi-allelically expressed genes and silenced genes (Fig. 5c). However, in a mouse embryo at blastocyst stage, the promoters of genes with bi-allelic expression show the highest CpG densities of all promoters of other types of genes (Fig. 5d).

Taken together, our data show that a significant proportion of genomic regions present distinct epigenomic patterns between maternal and paternal genomes in human early embryos. These patterns are involved in allelic expression. The allelically epigenetic features of orthologous regions or genes are not conserved between human and mouse early embryos.

## DISCUSSION

The question of how the allelic epigenomes are reprogrammed during human early embryonic development, and to what extent the characteristics of allelic epigenomes are conserved between humans and mice, is an important one. It has been reported that the H3K27me3 signal disappears at the eight-cell stage in human embryos, and re-established at the later stages [36,41]. In this regard, we only profiled H3K27me3 patterns of PG and AG embryos at the blastocyst stage. It is also reported that the open chromatin regions detected by DNase-seq and ATAC-seq are largely consistent. Moreover, the ATAC-seq data on mouse embryos at both the two-cell (ZGA stage in mice) and blastocyst stages, in which the parental alleles can be distinguished by SNPs, are available. Thus, we used the ATAC-seq data of mouse early embryos for comparison. Our data reveal that a significant portion of genomic regions show allelically epigenomic patterns in human early embryos. A larger portion of genomic regions in humans than in mice show parentally specific epigenetic states in early embryos. Furthermore, many allelic features are not conserved between humans and mice. For example, the allele-specific features of orthologous regions in human early embryos usually show no signals or bi-allelic signals in mice. Previous studies have reported mono-allelic bivalency at imprinted DMRs in mice [42,43]. Here, we also find that maternal DNA methylation and paternal H3K27me3 are associated with the repression of two alleles for imprinted or developmental genes. Besides, imprinted genes depending on the H3K27me3 in mice [12] do not have obviously allelic H3K27me3 patterns at human blastocysts. Given that non-canonical H3K27me3-dependent imprinting in mice occurs during early pre-implantation stages and global H3K27me3 are erased at the human eight-cell stage, we also guess it may be too late to observe H3K27me3-dependent imprinting in human AG and PG blastocysts. Therefore, it is advisable to focus future investigations on earlier-stage human embryos to explore this mechanism. In addition, our data indicate that the regions with allelic epigenetic states tend to be less conserved than those with bi-allelic epigenetic states in human early embryos. This may explain why the allelic epigenetic patterns are not conserved between humans and mice. It suggests that the regulatory mechanisms between human and mouse early embryogenesis are significantly different. Consistently, OCT4 regulates human ZGA but not in mice [38]. The epigenetic reprogramming between humans and mice also presents significant differences, such as high-order chromatin structure [6,7,44] and H3K27me3 patterns [34,36,40]. In humans, global H3K27me3 are erased at the eight-cell stage and reset at a later developmental stage [36,41]. Currently, more studies are needed to investigate the differences between humans and mice in terms of early embryogenesis.

Our data reveal that genome-wide DNA demethylation takes place in both human AG and PG embryos, and the DNA methylation levels of AG embryos are lower than those of PG embryos at several stages, which is consistent with previous findings that employed SNPs to track paternal and maternal DNA methylation in bi-parental embryos [29]. In addition, the parentally specific expression of many known imprinted genes and the parentally specific DNA methylation of many known ICRs or gDMRs have also been verified in the AG and PG embryos, as well as haploid cell lines [13,14]. The H3K27me3 signals were overall reversely correlated with DNA methylation levels in both AG and PG embryos, aligning with the results found in bi-parental embryos [41]. Collectively, these findings suggest that the epigenetic patterns of AG and PG embryos can represent the paternal and maternal epigenetic patterns of bi-parental embryos, respectively. It is noteworthy that a certain proportion of allelic DHSs are located in promoter regions and are more likely to play specific roles in developmental processes rather than fundamental cellular functions. Besides, it is also observed that a substantial number of parentally specific DHSs are located in the intergenic regions. Some of these cis-elements can be transcribed and function as putative enhancers. This finding indicates that cis-elements in humans could exhibit parentally specific characteristics, and may be associated with the parental contributions to allelic expression and embryo development. Moreover, we also find that promoters exhibit more allelic DHS signals in the eight-cell embryos than blastocysts, which suggests that the difference between maternal and paternal genomes in terms of chromatin accessibility in promoters becomes smaller during development.

Leng *et al.* also profiled the DNA methylome and transcriptomes of human PG and AG embryos [15]. However, due to the low coverage of their methylome data, they did not identify putative ICRs and novel imprinted genes. Our data provide a more comprehensive insight into the allelic features of epigenomes in human early embryos. This is a valuable resource when investigating the differences between paternal and maternal genomes in human early embryos. Because many imprinted genes are not expressed in human early embryos, the previously reported genes showing allelic expression in tissues are also integrated into the analysis. We finally detected five novel imprinted genes with gDMRs in their promoters, showing allelic expression in adult blood but no expression in human early embryos, such as *SLC46A2* and *RPS2P32* (Table S6) [23]. Unexpectedly, our data have identified a gDMR associated with *RPS2P32* (∼1.7k bp), which is inconsistent with a previous report that the associated DMR was methylated both in oocytes and sperm [45]. We next compared the gDMR of *RPS2P32* identified in our study with the PCR-validated *RPS2P32*-DMR [45]. We find that most regions of the gDMR could not be covered by the PCR-validated *RPS2P32*-DMR, indicating that the methylation level of most CpG sites of the gDMR remain unknown in that work [45]. Nevertheless, we still do no find evidence that the paternal allele of *RPS2P32*-DMR is hypermethylated in our data. Furthermore, it has been reported that the *RPS2P32*-DMR is also a repetitive region with multiple copies in the genome, which may have an impact on the accurate assessment of DNA methylation in this region due to other similar genomic regions. Additionally, one embryo and one SNP were used to track the paternal and maternal DNA methylation of *RPS2P32*-DMR. This suggests that discrepancies between that work and our study might be caused by limitations in the individual, or insufficient SNP. Therefore, a larger sample size and more comprehensive SNP information are required to ultimately address these disparities. Moreover, the authors in that work [45] also proposed that the presence of paternal hypermethylation might be attributed to their method, which was not able to rule out the co-enrichment of 5-hydroxymethylcytosine (5hmC) on the paternal allele in embryos.

Although epigenetic characteristics of human parental genomes are described in this study, several limitations should be stated. First, short-read sequencing used in this study makes it difficult to accurately investigate the epigenetic characteristics for some highly reparative genome elements, the association between the full-length isoform expression and epigenetics, and isoform-specific imprinting. Thus, it will be necessary to utilize long-read sequencing (such as ONT, PacBio) to address these challenges in human early embryos. Secondly, several findings in this study were mainly obtained in AG and PG uni-parental embryos and should be further confirmed in bi-parental embryos using more informative SNPs. For instance, the potential novel imprinted genes and gDMRs should be validated in bi-parental embryos and adult individuals. Furthermore, this study employs complete AG and PG blastocysts, which makes it impossible to discern the disparities between ICM and trophectoderm (TE). Due to the limited number of valuable human embryonic samples, this study solely concentrates on a few epigenetic traits. It would be important to expand our research by utilizing a larger number of human embryonic samples to delve into other allelic epigenetic traits such as H3K4me3, H3K27ac and H3K9me3.

## MATERIALS AND METHODS

Detailed materials and methods are available in the Supplementary Data. The regulatory framework pertaining to the utilization of human gametes and embryos for this research is according to the policies of the Human Biomedical Research Ethics Guidelines (set by National Health Commission of the People's Republic of China on December 1, 2016), the 2016 Guidelines for Stem Cell Research and Clinical Translation issued by the International Society for Stem Cell Research (ISSCR) and the Human Embryonic Stem Cell Research Ethics Guidelines (set by China National Center for Biotechnology Development on December 24, 2003). These policies and guidelines permit the use of human gametes and/or human embryos created or genetically manipulated in vitro, for scientific research purposes, provided that such usage is confined to a time frame of no more than 14 days. This study received approval from the Institutional Review Board of Reproductive Medicine of Shandong University (201810) with respect to its aims and protocols. The human gametes used in this study were donated by the patients under assisted reproductive therapy after they signed the informed consents. It was made explicitly clear to the donors that their decision to donate gametes would not in any way impede or hinder the progress of their ongoing therapy.

## Supplementary Material

nwad328_Supplemental_Files

## Data Availability

Data generated in this study have been deposited in the Genome Sequence Archive (GSA) with the accession number HRA000888. In addition, external data used in this study are listed below: DNA methylation of gametes, normal bi-parental embryos and haploid eight-cell in humans (GSA: CRA000114, HRA000888) and in mice (GSE56697); DNase-seq of haploid eight-cell in human (HRA000888); CUT&RUN of H3K27me3 in human oocyte and eight-cell (GSE124718), ChIP-seq of H3K27ac in human blastocyst (HRA002355), ChIP-seq of H3K27me3 in mouse embryos (GSE76687) and human sperm (GSE15594); RNA-seq in human haploid embryos (GSA: HRA000888) and in mouse embryos (GSE71434); ATAC-seq in mouse embryos (GSE66390). All codes are available upon reasonable request.
