# Supplementary material for: Allelic reprogramming of chromatin states in human early embryos
Source: Natl Sci Rev. 2024 Jan 2;11(3):nwad328. doi: 10.1093/nsr/nwad328 (PMC10917445; doi:10.1093/nsr/nwad328)
Supplement: nwad328_Supplemental_Files [file nwad328_supplemental_files.zip › suppmentary_Allelic_epigenomes_20231004.docx]

**Supplementary Materials for**

**Allelic reprogramming of chromatin states in human early embryos**

This file includes:

Materials and Methods

Figs. S1 to S6

References

Other Supplementary Materials include the following:

Supplementary Table 1 | The sample information of human embryos used in PBAT, ULI-NChIP-seq, and DNase-seq.

Supplementary Table 2 | Genes whose promoters exhibit AG-specific H3K27me3 and PG-specific DNA methylation.

Supplementary Table 3 | Maintained allelic DHSs from 8-cells to blastocyst stage and their overlapped gene promoters.

Supplementary Table 4 | Genes whose promoters are PG specifically DNA methylated.

Supplementary Table 5 | The status of parental DNA methylation in mouse regions that are homologous to human DMRs exhibiting PG-specific methylation.

Supplementary Table 6 | The gene imprinting information including the summary of reported allelic DMRs, the known imprinted genes with their pICRs, the novel imprinted genes, and the other genes whose promoters are overlapped with gDMRs.

**Materials and Methods**

**Ethics statement**

The regulatory framework pertaining to the utilization of human gametes and embryos for this research is according to the policies of the Human Biomedical Research Ethics Guidelines (set by National Health Commission of the People’s Republic of China on Dec. 1^st^, 2016), the 2016 Guidelines for Stem Cell Research and Clinical Translation issued by the International Society for Stem Cell Research (ISSCR) and the Human Embryonic Stem Cell Research Ethics Guidelines (set by China National Center for Biotechnology Development on Dec. 24^th^, 2003). These policies and guidelines permit the use of human gametes and/or human embryos created or genetically manipulated in vitro, for scientific research purposes, provided that such usage is confined to a time frame of no more than 14 days.

This study received approval from the Institutional Review Board of Reproductive Medicine of Shandong University (201810) with respect to its aims and protocols. The human gametes used in this study were donated by the patients under assisted reproductive therapy after they signed the informed consents. It was made explicitly clear to the donors that their decision to donate gametes would not in any way impede or hinder the progress of their ongoing therapy.

**Collection of human haploid embryos**

Parthenogenetic (PG) and androgenetic (AG) embryos were generated in accordance with previously described methods [1, 2]. No statistical methods were used to predetermine sample size. The immature oocytes at metaphase-I (MI) or germinal vesicle (GV) phase were donated by the patients who were receiving the in vitro fertilization (IVF) treatment. The immature oocytes were cultured in vitro to metaphase-II (MII) stage in IVM culture medium for 24 to 28 hours. The residual sperm obtained from the donors following their IVF treatment were collected and utilized for intracytoplasmic sperm injection (ICSI). The IVM oocytes were subjected to fertilization via ICSI, after which they were cultured in a time-lapse incubator (EmbryoScope, Vitrolife) for a period of 4-8 hours. The maternal and paternal pronuclei were discerned by means of time-lapse imaging, with the maternal pronucleus originating from the second polar body. The resulting zygotes were subsequently employed for the generation of haploid embryos.

To generate the androgenetic haploid embryos, the second polar body and the maternal pre-pronucleus were removed by using the Blastomere Bilpsy pipette (Sunlight Medical). Zygotes containing only the paternal genome were identified as human androgenic haploid embryos. These androgenic embryos were then cultured in G-1^TM^ PLUS medium (Vitrolife, 10128) (6% CO2, 5% O2, 37 °C) to 8-cell stage and then transferred into G-2^TM^ PLUS medium (10132, Vitrolife) for further culturing to blastocyst stage.

To generate the parthenogenetic haploid embryo, the paternal pronucleus was removed by using ICSI injection pipette (Sunlight Medical). Zygotes with only the maternal genome were identified as human parthenogenetic haploid embryos. These parthenogenetic embryos were then cultured in G-1^TM^ PLUS medium (6% CO2, 5% O2, 37 °C) until they reached the 8-cell stage. The embryos were then transferred to G-2^TM^ PLUS medium for further culture until they reached the morula stage or blastocyst (Day 5) stage. Following removal of the zona pellucida, high-quality AG and PG embryos were selected for further experimentation.

**ULI-NChIP-seq library preparation for H3K27me3**

Ultra-low-input micrococcal nuclease-based native ChIP and sequencing experiments (ULI-NChIP-seq) were performed as described with some modifications [3, 4]. Briefly, human haploid embryos collected in low-binding PCR tube were lysed in 30 μl cold lysis buffer (10 mM Tris-HCL pH 7.5, 10 mM NaCl, 3 mM MgCl2, 0.3% Triton X-100, 0.1% Na-deoxycholate, 1 mM PMSF, 1x Cocktail proteinase inhibitor) on ice for 20 minutes. Then, 20 μl MNase Master Mix were added, and incubated at 21 °C for 10 minutes. The reaction was stopped by adding 5.5 μl 100 mM EDTA and 4.5 μl 1% Triton X-100, followed by the incubation on ice for 30 minutes. Next, the volume of chromatin solution was adjusted to 220 μl by adding 160 μl 140mM ChIP RIPA buffer (10 mM Tris-HCL pH 7.5, 140 mM NaCl, 1 mM EDTA, 0.5 mM EGTA, 1% Triton X-100, 0.1% SDS, 0.1% Na-deoxycholate, 1 mM PMSF, 1x Cocktail proteinase inhibitor). The chromatin solution was rotated at 4 ^o^C for 1 hour. After vortexing for 30 seconds, 20 μl of chromatin solution was used as input, the remaining 200uL of chromatin solution was used for immunoprecipitation assay. 10 μl of Dynabeads Protein A beads (Life Technologies, 10001D) was washed twice with 150 μl ice-cold 140 mM ChIP RIPA buffer, and then resuspended in 150 μl of 140 mM ChIP RIPA buffer. After adding 1 µL of H3K27me3 antibody (Active Motif, 39155), the bead suspension was incubated at 4°C for at least 2.5 hours on a rotator. The antibody-coated beads were washed twice with 200 μl 140 mM ChIP RIPA buffer, and then incubated with 200uL chromatin solution for immunoprecipitation at 4 °C overnight. After that, the beads were washed once with 250 mM ChIP RIPA buffer (10 mM Tris-HCL pH 7.5, 250 mM NaCl, 1 mM EDTA, 0.5 mM EGTA, 1% Triton X-100, 0.1% SDS, 0.1% Na-deoxycholate, 1 mM PMSF, 1x Cocktail proteinase inhibitor), twice with 500 mM ChIP RIPA buffer (10 mM Tris-HCL pH 7.5, 500 mM NaCl, 1 mM EDTA, 0.5 mM EGTA, 1% Triton X-100, 0.1% SDS, 0.1% Na-deoxycholate, 1 mM PMSF, 1x Cocktail proteinase inhibitor), and once with TE buffer (10 mM Tris-HCl pH 8.0, 1 mM EDTA). To release the chromatin DNA, the beads were incubated in 100 μL of ChIP elution buffer (10 mM Tris-HCl pH8.0, 5 mM EDTA, 300 mM NaCl, 0.5% SDS) containing 5 µL of proteinase K (Qiagen, 20 mg/ml stock) at 55 °C for 6 hours. 180 μl of SPRIselect beads (1.8X volume) were used to purified the chromatin DNA. The DNA libraries were constructed by using NEBNext Ultra II DNA Library Prep Kit for Illumina (NEB, E7645S) according to manufactory’s instruction. Lastly, the DNA fragments of sizes from 300 to 700 bp were purified by using SPRIselect beads, and then eluted in 15 μL water. The libraries were sequenced as 150 bp paired-end on the Illumina HiSeq X-Ten instrument. Two haploid blastocysts were used for an ULI-NchIP-seq assay. Two biological replicates were carried out for PG and AG embryos (Supplementary Table 1).

**PBAT library preparation**

PBAT experiments were performed as described with some modifications [5-7]. In brief, human haploid embryos were initially lysed in the lysis buffer (20 mM Tris-HCl pH 8.0, 2 mM EDTA, 20 mM KCl, 2 mg/ml proteinase K) at 56 ^o^C for 1.5 hours, followed by heat inactivation at 75 ^o^C for 30 min. Bisulfite treatment was then performed by using EZ DNA Methylation-Gold Kit (Zymo Research, D5006) according to manufactory’s instruction. Subsequently, the first-strand DNA for bisulfite treated DNA was synthesized using 75 U of Klenow Fragment (3´-5´ exo-) (NEB, M0212M) and the biotinylated random primer BioPEA_N4_37 (5′-biotin-ACA CTC TTT CCC TAC ACG ACG CTC TTC CGA TCT NNN N-3′) through five cycles of random priming and extension. Excessive primers were then removed by incubating with 40 U of Exonuclease I (NEB, M0293S) at 37 ^o^C for 1 hour, followed by DNA purification using 1 volume of SPRIselect beads. The biotinylated DNA was captured using Streptavidin beads, and the second-strand DNA was synthesized using 75 U of Klenow Fragment (3’-5’ exo-) with another random primer, 2.0-N (5’-GTG ACT GGA GTT CAG ACG TGT GCT CTT CCG ATC TNN NN-3’). Then, The DNA fragments captured by Streptavidin beads were subjected to amplification via 10-15 cycles of PCR using primers designed for the Illumina TruSeq DNA libraries. Following amplification, DNA fragments ranging in size from 300 to 700 bp were purified using SPRIselect beads. Subsequently, the libraries were sequenced as 150 bp paired-ends on the HiSeq X-Ten instrument (Illumina). Each PBAT library was generated utilizing one or two haploid morulae, or one haploid blastocyst. At least two biological replicates were conducted for both PG and AG embryos (Supplementary Table 1).

**DNase-seq library preparation**

DNase-seq experiments were conducted according to a previously described protocol, with some modifications [8]. Briefly, embryos were lysed in 40 μL of cold lysis buffer (10 mM Tris-HCl pH 7.5, 10 mM NaCl, 3 mM MgCl2, 0.5% Triton X-100) for 30 minutes on ice. Subsequently, 10 μL of diluted DnaseI (Roche, 04716728001, LOT:34944500) was added to the final concentration of 150 U/ml and incubated at 37°C for 5 minutes. Reaction was halted by adding 50 μL stop buffer (10 mM Tris-HCl pH 7.5, 10 mM NaCl, 0.2% SDS, 20 mM EDTA) supplemented with 40 μg Proteinase K (Qiagen, 19133) and incubated at 55°C for 1 hour. After adding carrier RNA (Tiagen, RT416-02), the DNA was purified by Zymo Oligo Clean Concentrator (Zymo Research, D4060) and then eluted in 50 μL TE (2.5 mM Tris-HCl pH 7.5, 0.05 mM EDTA). DNA libraries were generated using the NEBNext Ultra II DNA Library Prep Kit for Illumina (NEB, E7645S) as per the manufacturer’s instructions. Following 8 cycles of PCR amplification, DNA fragments ranging in size from 150-400 bp were selected with 0.7 volume plus 0.7 volume of SPRIselect beads (Beckman Coulter, B23318). The DNA products were amplified with another 7 cycles of PCR and purified using 1.3 volume of SPRIselect beads. The libraries were sequenced as 150 bp paired-ends on the HiSeq X-Ten platform (Illumina). For a DNase-seq library, one haploid blastocyst was used. Two biological replicates were carried out for PG and AG embryos. (Supplementary Table 1).

**H3K27me3 ULI-NchIP-seq data analysis**

The ULI-NchIP-seq raw reads were cropped to 100 bp, and the low-quality reads were removed using Trimmomatic [9]. Paired reads were mapped to the human genome (version hg19) by Bowtie2 v2.2.9 [10]. The reads with low mapping qualities (MAPQ <10) were discarded, and the PCR duplicates were removed by using Picard v2.18.25. The reads for two biological replicates were merged to call peaks by MACS2 v2.1.0 [11] with parameters “–SPMR –broad”. H3K27me3 peaks (domains) were further filtered based on the signal enrichment in the ChIP sample comparing to that in the input. The fold enrichment for the H3K27me3 peaks against random Poisson distribution with local lambda should be >= 2.5. The normalized signals of H3K27me3 were generated by using bdgcmp tool in MACS2 with the parameter “-m FE”. ChIP-seq signal was visualized by Integrative Genomics Viewer. The H3K27me3 signal for a specified genomic region was calculated by the mean value of H3K27me3 signal in the region, which was calculated by using bwtools with the bigwig file of H3K27me3 signal. The Pearson correlation coefficient I of tag densities in the genome-wide 5 kb bins between two biological replicates was calculated to evaluate the reproducibility.

**PBAT DNA methylome data analysis**

The analysis of PBAT DNA methylome data was performed according to the methods outlined in the previous study [7]. In brief, the sequencing reads were processed using Trimmomatic to eliminate adapter sequences and low-quality reads [9]. Paired reads were mapped to the human genome (version hg19) by using Bismark_v0.20.0 [12]. The paired reads failed to align to human genome were re-aligned to the genome in the single-end mode. Duplicated reads were removed via deduplicate_bismark tool in Bismark. Overlapping regions in the genome between paired-end reads were clipped from one read using the clipOverlap function in bamUtil. The CpG methylation level (ML) for each CpG site was determined by combining both paired-end and single-end alignments, and only CpG sites with a read depth greater than or equal to three were retained for subsequent analysis. The reproducibility of the data between two biological replicates was evaluated using the Pearson correlation coefficient (r) of CpG methylation in 20 kb bins included at least 40 CpGs.

**Quantification of methylation levels of CpGs**

The CpG site i was characterized by two counts: m_i_, representing the number of reads displaying methylation at cytosines (methylated Cs) on both strands, and u_i_, representing the number of reads displaying unmethylation at cytosines (unmethylated Cs) on both strands. The methylation level of the CpG site i was then estimated as m_i_/(m_i_ + u_i_).

**Quantiﬁcation of average CpG methylation levels of different genomic elements**

The methylation level of a genomic element was determined by calculating the ratio of the number of methylated Cs to all of the methylated and unmethylated Cs in the genomic element. Promoters are referred to the regions from 1 kb upstream to 1kb downstream of TSSs (transcriptional start sites). Only the promoters with at least 5 different CpG sites were covered by sequencing reads were considered for further analysis. For other genomic elements, a minimum requirement of 3 different CpG sites covered by sequencing reads was considered for their inclusion in subsequent analyses.

**Identification of the differentially methylated sites/regions (DMSs/DMRs) between AG and PG embryos**

The CpG sites with read depth >= 5 were considered for statistical test. The differences of the methylation levels of CpG sites between two samples was evaluated by a two-tailed Fisher’s Exact Test with p-values being adjusted via the Benjamini and Hochberg method [13]. The CpG sites with the adjusted p values < 0.1 were defined as DMSs. In the absence of any specification, all DMSs were required to exhibit methylation level differences between two samples greater than 0.2. Subsequently, maternal DMSs (higher CpG methylation in PG embryos) were merged into maternal pre-DMRs if the distances between adjacent maternal DMSs were less than 500 bp. Paternal DMSs (higher CpG methylation in AG embryos) were merged into paternal pre-DMRs if the distances between adjacent paternal DMSs were less than 500 bp. Next, the maternal and paternal pre-DMRs were combined to form pre-DMRs. The final DMRs were obtained if the pre-DMRs contained at least three DMSs, adjusted p values < 0.1, the methylation level differences of pre-DMRs must be greater than 0.2, and the regions size were larger than 50bp.

**Identification of the germ line DMRs related with gene imprinting**

The DMSs between AG and PG haploid blastocysts were identified as described above. Then, maternal DMSs were the DMSs that satisfied the following criteria: i. The MLs of DMSs were higher in PG blastocyst comparing to AG blastocyst; ii. the MLs of DMSs in sperm should be <=0.35; iii the ML differences of DMSs between oocyte and sperm (ML_oocyte_–- ML_sperm_) should be >=0.5. These maternal DMSs were merged into large regions if the distances between adjacent DMSs were less than 500 bp, and the regions were defined as maternal pre-iDMRs. Paternal DMSs were the DMSs that satisfied the following criteria: i. The MLs of DMS were higher in AG blastocyst comparing to PG blastocyst; ii the ML differences of DMSs between sperm and oocyte (ML_sperm_-ML_oocyte_) should be >= 0.5. These paternal DMSs were merged into large regions if the distances between adjacent DMSs were less than 500bp, and the regions were defined as paternal pre-iDMRs. Next, the maternal and paternal pre-iDMRs were collected together to form a list of pre-iDMRs. The pre-iDMRs were defined as iDMRs and kept for further analysis if they meet the following criteria: i. The pre-iDMRs must contain at least 3 DMSs; ii. The absolute values of ML differences of pre-iDMRs between AG and PG haploid blastocysts should be >= 0.2; iii. The adjusted p values of the ML differences of pre-iDMRs between AG and PG haploid blastocysts should be < 0.1. Furthermore, the methylation levels of iDMRs were calculated in human gametes and embryos. The iDMRs were referred as germline DMRs (gDMRs) and retained for further analysis if they meet the following criteria:

1. The absolute values of ML differences of iDMRs between oocyte and sperm should be >= 0.5;
2. The absolute values of ML differences of iDMRs between PG and AG blastocysts should be >= 0.5;
3. The absolute values of ML differences of iDMRs between PG and AG embryos at morula or 8-cell stages should be >= 0.5;
4. The MLs of iDMRs in human diploid embryos at two or three stages, including 8-cell, morula and blastocyst stages, should be between 0.35 and 0.65.
5. The MLs of iDMRs in human placenta or 6-week embryo should be between 0.35 and 0.65.

The gDMRs near imprinted genes were manually inspected. These gDMRs would be defined as placenta-specific if they exhibited intermediate DNA methylation exclusively in placenta. The gDMRs would be defined as embryo-specific if they exhibited intermediate DNA methylation exclusively in 6-week embryo. The gDMRs would be defined as non-specific if they exhibited intermediate DNA methylation in both the placenta and 6-week embryo.

**DNase-seq data analysis**

The analysis of DNase-seq data was performed according to the methods described in the previous study [7]. The sequencing reads were cropped to 100 bp from 3’ end and trimmed by using Trimmomatic to remove adapter sequence and reads with low qualities [9]. All Reads 1, and Reads 2 from unpaired reads were aligned to human genome hg19 by Bowtie v1.2.0 [14] with parameter “-m 1”. Any PCR duplicated reads were subsequently eliminated using Picard v2.18.25. DHSs were identified via a hotspot algorithm with a FDR threshold of less than 0.01 [15]. The FPKM (Fragment Per Kilobase per Million mapped reads) value was computed for each non-overlapping 5 kb genomic window (bin), serving as a measure of tag density for the DNase-seq data. Data reproducibility between two biological replicates was assessed by determining the Pearson correlation coefficient (r) of tag densities within 5 kb bins. DHS peaks were called by merging two biological replicates. For the creation of a DHS master list, we concatenated DHSs in PG and AG embryos at 8-cell and blastocyst stages, the overlapped DHSs were merged into a large DHS. DHSs in the master list that overlapped with original DHSs in PG or AG embryos were further analyzed as DHSs in those samples. The FPKM values of DHSs in each sample were calculated. The tracks of DNase-seq signal visualized in Integrative Genomics Viewer (IGV) were generated by bamCoverage in Deeptools2 suite with paramete– “--noralizeUsingR–M --extendReads 150” [16-18]. Owing to the varying signal-to-noise ratios between PG and AG samples at identical developmental stages, the sequencing depth-normalized DHS signal was not suitable for direct use in calling allelic DHSs between PG and AG samples. As described in prior research [19], we assumed that the top 10% DHSs (5,000-10,000 DHSs) should exhibit similar signal levels between PG and AG samples. Accordingly, we calculated the scale factor as the ratio of the median signal level in the top 10% DHS peaks between PG and AG samples. Each DHS's FPKM value was then adjusted by multiplying it with this scale factor to ensure comparability. After scale factor normalization, DHSs solely observed in AG embryos and showed signal fold change (AG/PG) more than 2 were defined as AG-specific DHSs, vice versa were PG-specific DHSs.

**RNA-seq data analysis**

The analysis of published RNA-seq data of human haploid embryos was performed according to the methods described in the previous study [7].

**Epigenetics signal on human genome**

The human genome was divided into continuous 300 bp bins. The bins were classified into 4 groups according to their DHS and H3K27me3 signal. The bins in group I were only overlapped with DHS in any haploid embryos. The bins in group II were overlapped with both DHS and H3K27me3 peak in any haploid embryos. The bins in group III showed were overlapped with only H3K27me3 peaks in any haploid embryos. The bins in group IV showed neither DHS nor H3K27me3 signal in any haploid embryos. The bins in each group were merged into large genomics regions if the bins are next to each other. Next, the DHS signal, H3K27me3 signal and CpG methylation levels (CpG ML) in the four groups of genomic regions were calculated. These regions were sorted by the differences of CpG MLs between AG and PG blastocysts in each group (Fig. 1b). The regions will be ignored if less than three CpGs were covered by sequencing reads at any stages. The DHSs signal were normalized by Z-score in each column (sample). The genomic regions with ML(AG-PG) >=0.5 were defined as AG-specific methylated regions, while the regions with ML(PG-AG) >=0.5 were defined as PG-specific methylated regions. The regions marked with AG-specific H3K27me3 are defined under two criterions: 1) H3K27me3 signal of the regions in AG is at least 1.5-fold higher than PG; 2) H3K27me3 signal of the regions in AG is at least 2; 3) H3K27me3 signal of the regions in PG is at less than 1.5, vice versa were PG-specific H3K27me3 regions.

**Promoters with AG and PG specifically epigenetic signal in human early embryos**

The promoters with ML(AG-PG)>=0.5 were defined as AG specifically DNA methylated, vice versa were PG specifically methylated promoters. The promoter was thought to be marked with AG-specific H3K27me3 when the promoter overlapped with AG H3K27me3 peak, and the H3K27me3 signal of AG promoter was large than 1.5, as well as at least 1.5-fold than PG promoter whose signal was less than 1.5, vice versa were the promoters with PG-specific H3K27me3. The promoters that overlapped with AG-specific DHSs but not with PG DHSs were consider as promoters with AG-specific DHSs, vice versa were the promoters with PG-specific DHSs.

**phastCons analysis**

The files about the phastCons scores for human genome were downloaded from UCSC genome browser. The phastCons score of a specified region was calculated by the average phastCons score of all sites in this region. Briefly, to evaluate the conservation of cis-elements among different species during evolution, the 100-way vertebrates phastCons scores were used, which represent the conservation of each base pair according to the sequence alignment of 100 species, including primate subset, mammal subset, fish subset, and so on. Higher phastCons score represents more conservation. The phastCons score for each cis-element was calculated by the mean phastCons score of all the bases in the cis-element.

**Allelic ATAC-seq, H3K27me3 and DNA methylation patterns in mouse early embryos**

To analyze the ATAC-seq, H3K27me3 and DNA methylation patterns of parental genomes in mouse early embryos, we downloaded the raw data and processed data from GSE66390, GSE76687 and GSE56697, respectively. The SNP information among common mouse strains for mm10 genome version is downloaded from Mouse Genomes Project. SNPsplit (v0.3.2) was used to distinguish maternal and paternal reads in ATAC-seq data for 2-cell and inner cell mass (ICM) (GSE66390). For each ATAC-seq peak covered by reads with parental SNPs, we counted the numbers of reads from paternal and maternal genomes, respectively. The reads numbers were then checked by Binomial test. For a bi-allelic ATAC-seq peak, the probabilities of a sequencing read from paternal genome or maternal genome are both 0.5. An ATAC-seq peak with binomial test p value < 0.05 and the fold change of reads numbers between parental genomes > 2 was considered as an allelic peak. The paternal and maternal H3K27me3 of mouse ICM were obtained from the processed data in GSE76687. Maternally specific H3K27me3 peaks were those maternal H3K27me3 peaks that not overlapped with paternal H3K27me3 regions, while the paternally specific H3K27me3 peaks were those paternal H3K27me3 peaks that not overlapped with maternal H3K27me3 regions. The promoter was thought to be marked with maternal specific H3K27me3 if the promoter only overlapped with maternal H3K27me3 peak, and the sum of H3K27me3 signal of each covered sites in maternal promoter was at least 2-fold than the paternal, vice versa were promoters with paternal specific H3K27me3. The paternal and maternal DNA methylation data of gametes, 2-cell, 4-cell and ICM were obtained from the processed data in GSE56697. For blastocyst (E3.5 embryo), SNPsplit was used to distinguish maternal and paternal DNA methylation reads. The genomic bins (300bp) with at least two CpG covered by sequencing reads in were used to calculated the average methylation levels of maternal and paternal in 2-cell, 4-cell and blastocyst. For sperm or oocyte, genomic bins (300bp) with at least three CpG were used. The promoters with at least three CpGs covered by sequencing reads in maternal or paternal genome were considered for further analysis. To explore the relationship between allelic H3K27me3 and DNA methylation of ICM in same genome version, we realigned DNA methylation data of ICM to mm9 genome version and then obtained maternal and paternal DNA methylation reads by using SNPsplit. If no specifications were provided, the genomic regions exhibiting parentally specific methylation were defined based on the aforementioned criteria.

**Allelic RNA analysis in mouse embryos**

To analyze the gene expression pattern parental genomes in mouse early embryos, we downloaded the RNA-seq data from GSE71434. Additionally, SNP information among common mouse strains for the mm10 genome version was obtained from the Mouse Genomes Project. SNPsplit was then used to distinguish maternal and paternal reads in RNA-seq data for mouse ICM. For each gene covered by reads with parental SNPs, we counted the numbers of reads from paternal and maternal genomes, respectively. The read numbers were then subjected to a Binomial test. For a gene with bi-allelic expression, the probabilities of a sequencing read from paternal genome or maternal genome are both 0.5. A gene with binomial test p value < 0.001 (BH adjusted p value <0.05) and the fold change of reads numbers between parental genomes > 2 was deemed to be allelically expressed.

**Comparison of homologous genome regions with parentally specific epigenetic signal between human and mouse**

To explore whether the allelic DHSs and DMRs are conserved between human and mouse, we firstly obtained the homologous regions of human allelic DHSs and DMRs in mouse by using LiftOver tool with “-minMatch=0.9”. Then, we calculated the maternal and paternal ATAC signal and DNA methylation on these homologous regions. For the homologous DMRs with parentally specific methylation, the DNA methylation difference between two alleles should be large than 0.5.

**The gene imprinting**

The known imprinted genes were from this review [20-22], and several genes that could not found in genome annotation files (hg19 version) were not included. The gDMRs which were closest to the known imprinted genes were regarded as their putative germ line ICRs (pICR) if the distances between gDMRs and genes were less than 1 Mb. For new imprinted genes which were not reported in a list of known imprinted genes, we regarded the genes, which showed the three-fold changes of gene expression between AG and PG embryos at both morula and blastocyst, as novel imprinted gene in human early embryos. Besides, the novel imprinted gene must harbor at least one gDMR within 200kb, based on the fact that many known imprinted genes harbored gDMRs in the promoters or promoters nearby. We then identified genes whose promoters had gDMRs and were not included in the list of known and novel imprinted genes. The reported (known) allelic DMRs were obtained from these studies [21-26]. For some reported allelic DMRs [21, 25] with mild DNA methylation level difference between PG and AG embryos, we only considered the allelic DMRs whose methylation differences were larger than 0.25. For known imprinted genes, we regarded those genes with the fold change of gene expression between AG and PG blastocysts >= 3 as parentally specific imprinted genes. Those known imprinted genes with FPKM values of RNA expression < 1 in both AG and PG blastocysts were referred as non-expressed imprinted genes. The rest of imprinted genes were expressed in human embryos, but did not show allelically specific expression.

**Annotation files for genomic elements**

The hg19 refGene files downloaded from UCSC Table Browser were used for genome annotations. The annotation files for genomics elements, including promoters, exons and introns, were download from UCSC Table Browser.

**Statistical analysis**

Statistical analyses and plots were implemented with R (3.4.4) (http://www.r-project.org). Pearson or Spearman Correlation Coefficients were calculated by using the ‘cor’ function. Wilcoxon signed-rank test was performed by using ‘wilcox.test’ function (two.sided). Hypergeometric test was performed by using ‘phyper’ function. Fisher’s Exact Test was performed by using the ‘fisher.test’ function with default parameters (two.sided). BH-adjusted p values were calculated by ‘p.adjust’ function with the parameter “method=‘BH’”.

**Supplementary Figures**

**
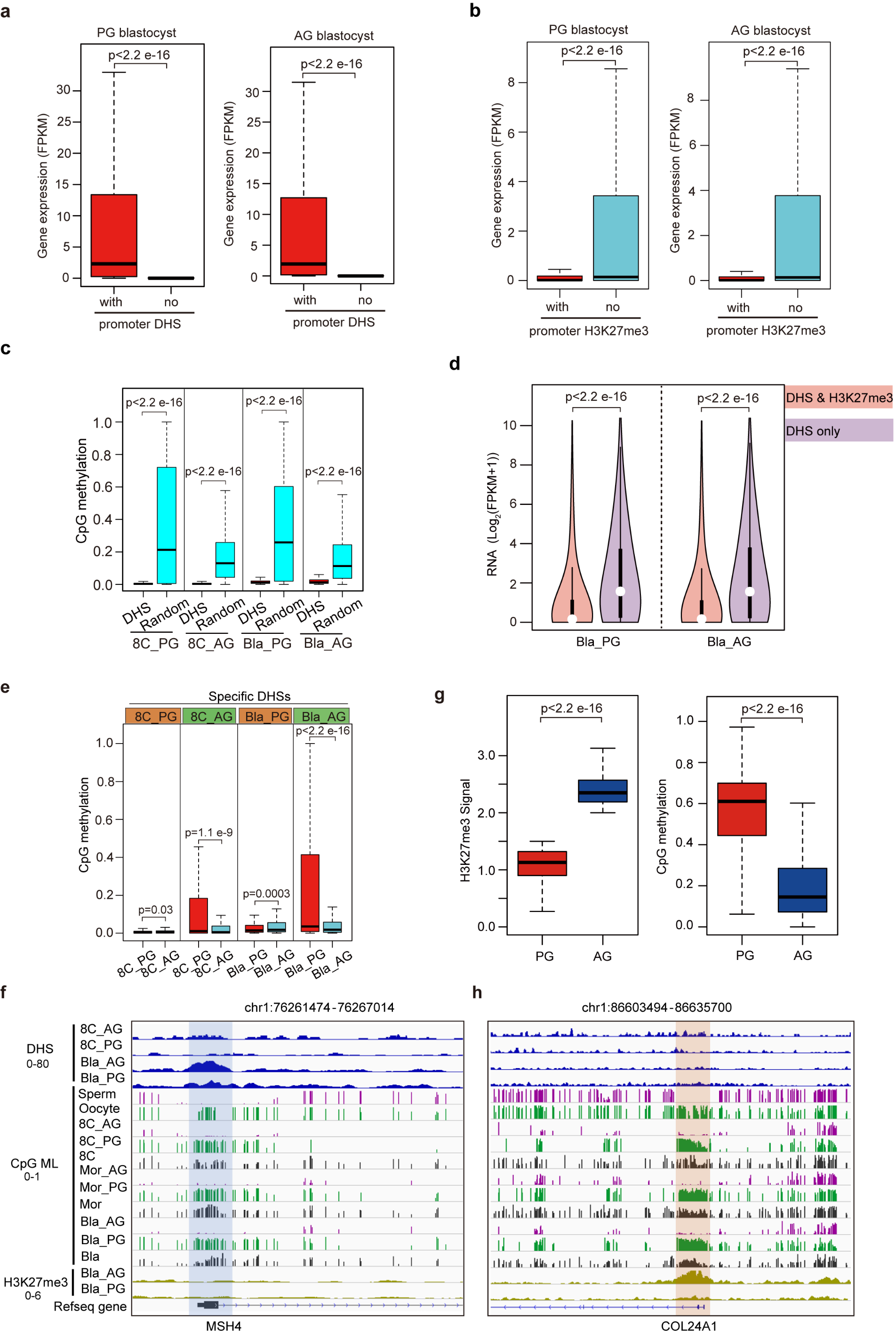
**

**Supplementary Fig. 1 | The relationship among epigenetic modifications and gene expression in human early embryo. a,** Boxplot comparing the RNA expression levels of the genes with promoter DHSs and those without promoter DHSs in human PG and AG blastocysts. Wilcoxon rank sum test was used. **b,** Box plot comparing the RNA expression levels between the genes with promoter H3K27me3 and those without promoter H3K27me3 in PG and AG blastocysts. Wilcoxon rank sum test was used. **c,** Boxplot comparing the DNA methylation levels of DHSs and random regions in human haploid embryos. Wilcoxon rank sum test was used. **d,** Violin plot comparing the RNA expression levels of genes whose promoters harbor both DHS and H3K27me3 signal (belong to group II in Fig. 1**b**) or only DHS signal (belong to group I in Fig. 1**b**), in PG and AG blastocysts. Wilcoxon rank sum test was used. **e,** Box plots comparing the DNA methylation levels of the AG or PG-specific DHSs in AG and PG embryos. Wilcoxon rank sum test was used. **f,** Genome browser view of DHS signal, CpG methylation levels and H3K27me3 signal at MSH4 loci in human gametes and embryos. **g,** Box plots showing the H3K27me3 signal (left) and CpG ML (right) of the regions (group II and group III in Fig 1**b**) with AG-specific H3K27me3 in PG and AG blastocysts. Wilcoxon rank sum test was used. **h,** Genome browser view of DHS signal, CpG methylation levels and H3K27me3 signal at COL24A1 loci in human gametes and embryos.


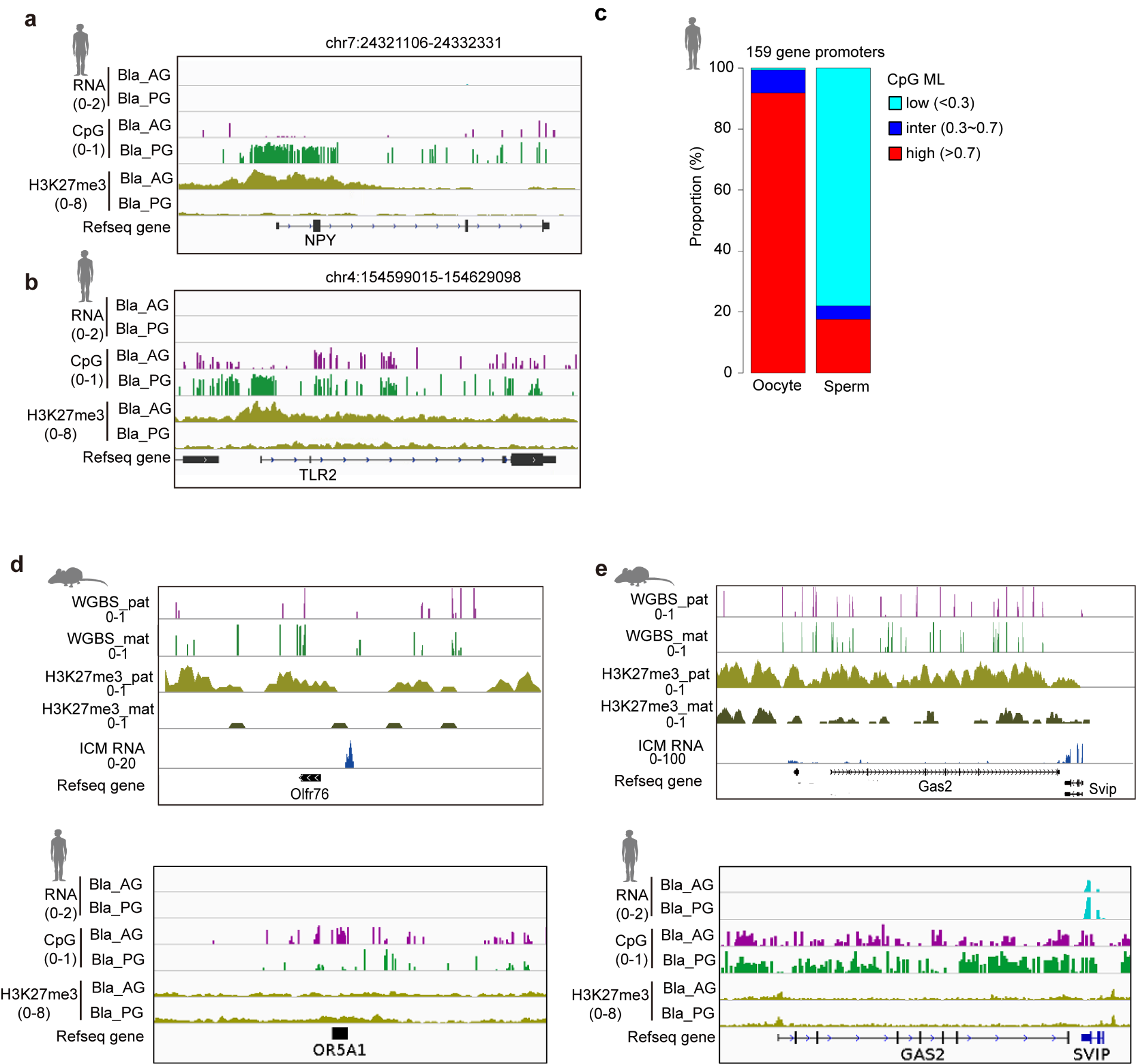


**Supplementary Fig. 2 | The parentally specific H3K27me3 signal in human embryos. a-b,** Genome browser view of RNA expression, DNA methylation and H3K27me3 signal at NPY (**a**) and TLR2 (**b**) loci in human AG and PG blastocysts. **c,** Plots showing the DNA methylation levels of the promoters with both PG-specific DNA methylation and AG-specific H3K27me3 in human sperm and oocyte. The proportions of the promoters with different CpG methylation levels are shown. **d-e,** Genome browser view of RNA expression, parental DNA methylation and parental H3K27me3 signal for mouse genes Olfr76 (**d**) and Gas2 (**e**) at blastocyst stage, alongside their homologous genes OR5A1 (**d**) and GAS2 (**e**) in human.

**
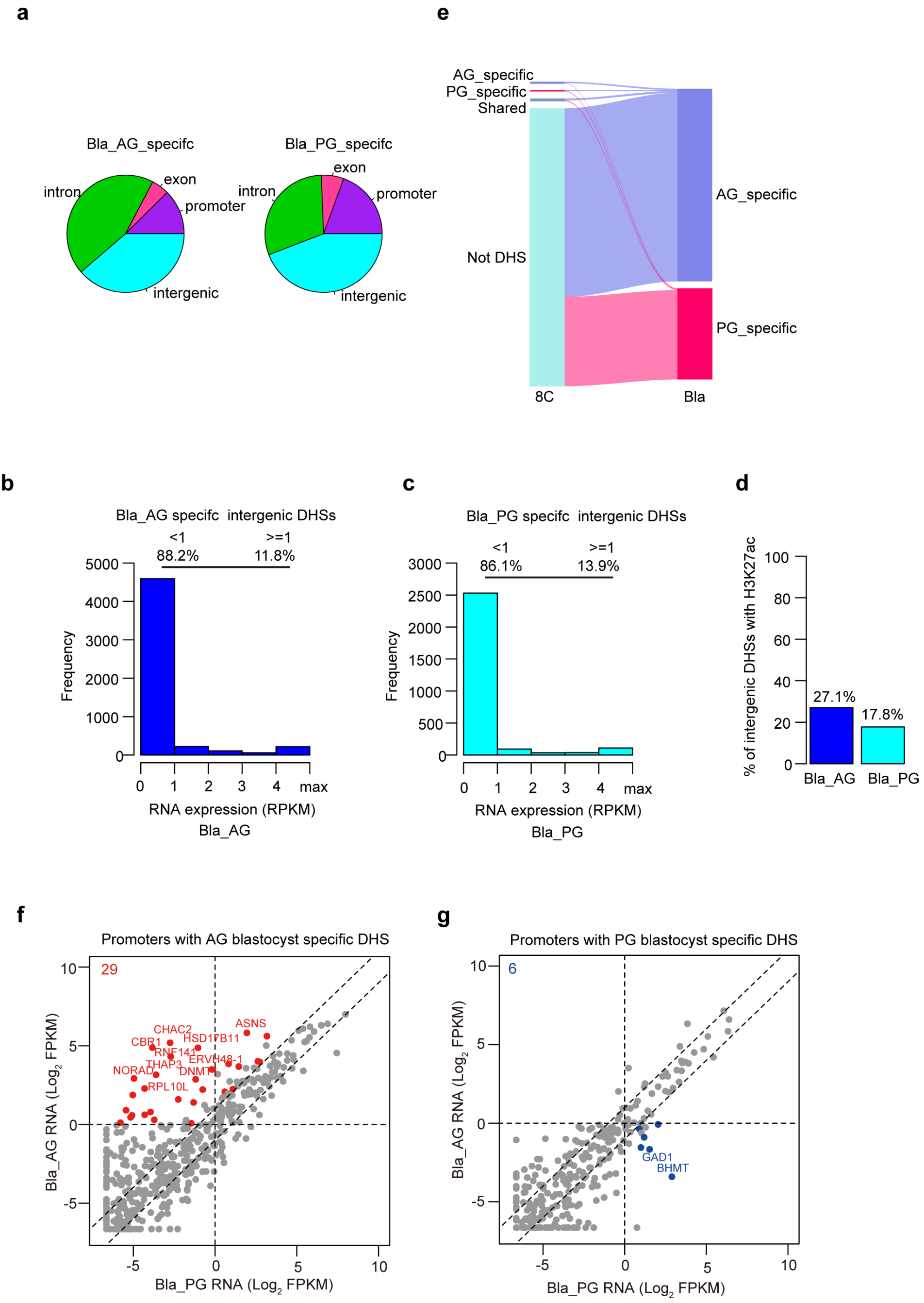
**

**Supplementary Fig. 3 | Parentally specific chromatin accessibility in human PG and AG embryo.** **a,** Pie chart comparing the genomic distribution of AG and PG-specific DHS at blastocyst stages. **b-c,** Bar plot showing the number (Frequency) of blastocyst AG-specific intergenic DHSs (**b**) or PG-specific intergenic DHSs (**c**) with different RNA expression (RPKM). **d,** Plot illustrating the proportion of blastocyst AG and PG-specific intergenic DHSs that overlap with H3K27ac peaks in blastocysts. **e,** Plot displaying the chromatin status of AG blastocyst specific DHSs and PG blastocyst specific DHSs at the 8-cell stage. The chromatin status of DHSs includes AG 8-cell specific DHS, PG 8-cell specific DHSs, bi-allelic DHSs (shared) and not DHSs at both in AG and PG 8-cell. **f-g,** Scatter plot showing RNA expression levels of the genes whose promoters harbor blastocyst AG-specific DHSs (**f**) or blastocyst PG-specific DHSs (**g**) in human PG and AG embryos. The number of the genes with AG-specific DHSs is shown with red in (**f**). The red dots in (**f**) represent the genes with AG-biased expression. The number of the genes with PG-specific DHS is shown with blue in (**g**). The blue dots in (**g**) represent the genes with PG-biased expression.

**
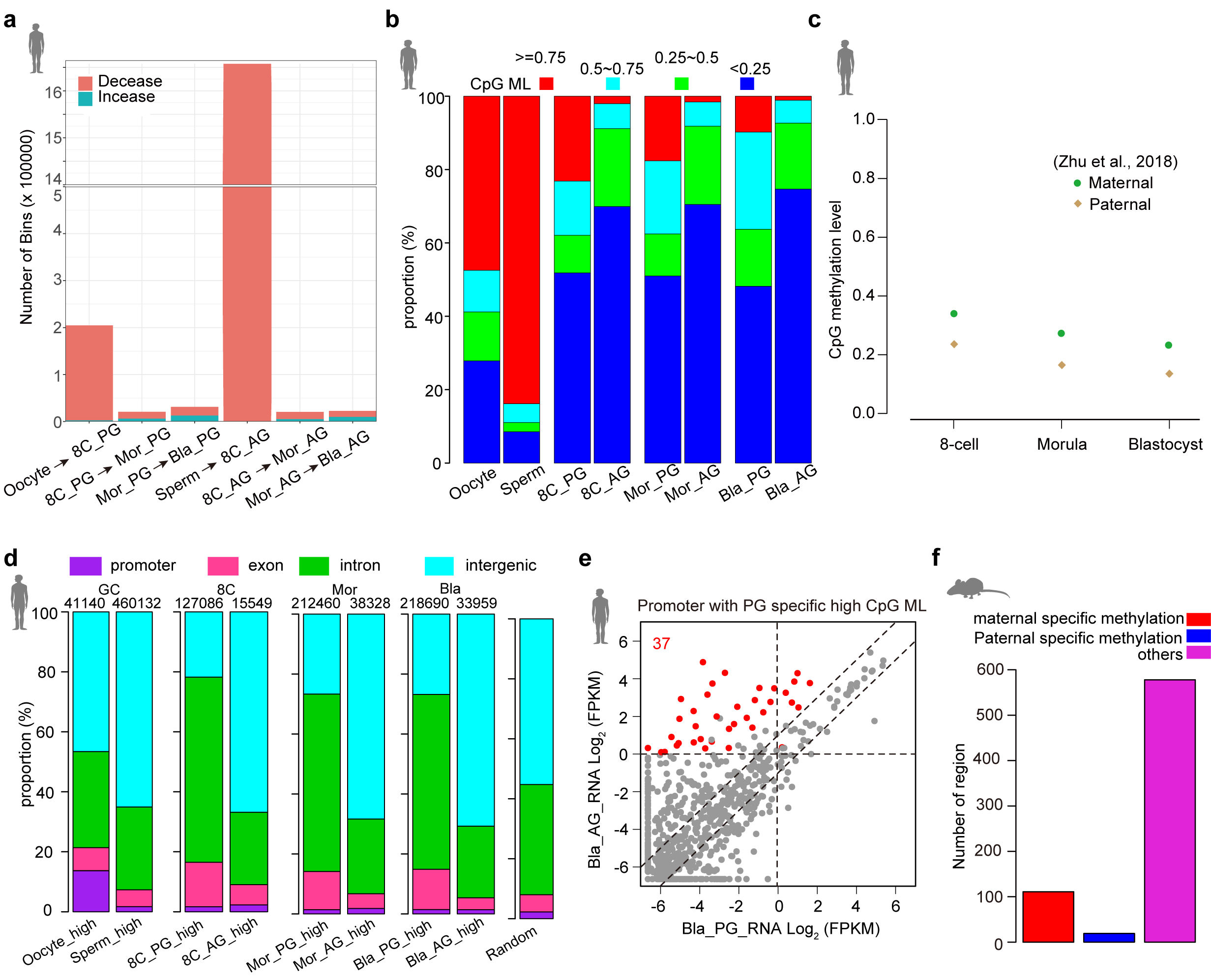
**

**Supplementary Fig. 4 | DNA methylomes in human PG and AG embryos.** **a,** Bar plot showing DNA methylation dynamics of maternal and paternal genomes between consecutive stages in human. The genome is binned into continuous 300 bp regions to calculate their DNA methylation levels. The 300 bp regions will be included in which the DNA methylation difference was at least 0.5 between two stages. **b,** Plots showing the proportions of genomic regions with different CpG methylation levels (ML) in human gametes, haploid PG and AG embryos. **c,** CpG DNA methylation dynamics of the paternal and maternal genomes in human early embryos. The DNA methylome data are from (Zhu et al., 2018), in which the parental genomes can be distinguished by SNPs. **d,** Genomic distribution of the DMRs between paternal genome and maternal genome in human gametes and haploid embryos. GC represents gametes. A set of random regions matching the length and number of total DMRs in human PG and AG blastocyst is plotted as control. **e,** RNA expression levels of the genes, whose promoters harbor PG-specific hypermethylation at blastocyst stage, in human PG and AG blastocysts. The red dots represent the genes with AG specifically high expression. The number of the genes with AG specifically high expression is shown in red. **f,** Bar plot summarizing the DNA methylation levels of the orthologous regions in mouse blastocyst, which harbor PG-specific DNA methylation in human blastocyst (PG specifically methylated DMRs).

**
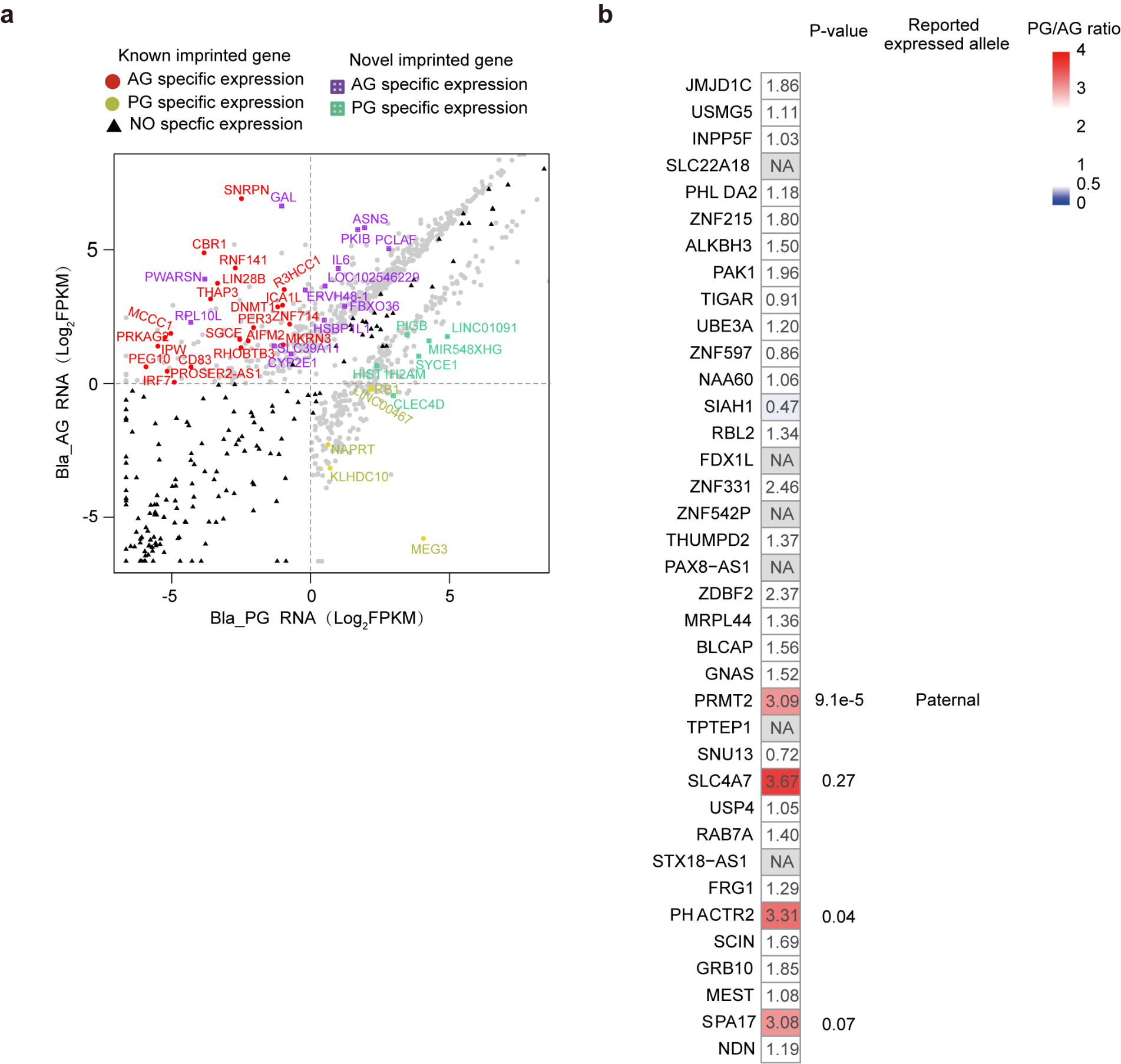
**

**Supplementary Fig. 5 | Imprinted gene in human.** **a,** Scatter plot showing the expression levels of imprinted genes and DEGs in human PG and AG blastocysts. The imprinted genes are labeled. **b,** Heatmap showing the ratio of the gene expression levels in PG morula to the gene expression levels in AG morula. The published RNA-seq data from (Leng et al., 2019) are used for this analysis. The ratio values are shown. ‘NA’ means that the expression of the gene is not detected. PRMT2 is reported to be a paternally expressed gene. The P values of t test are shown.

**
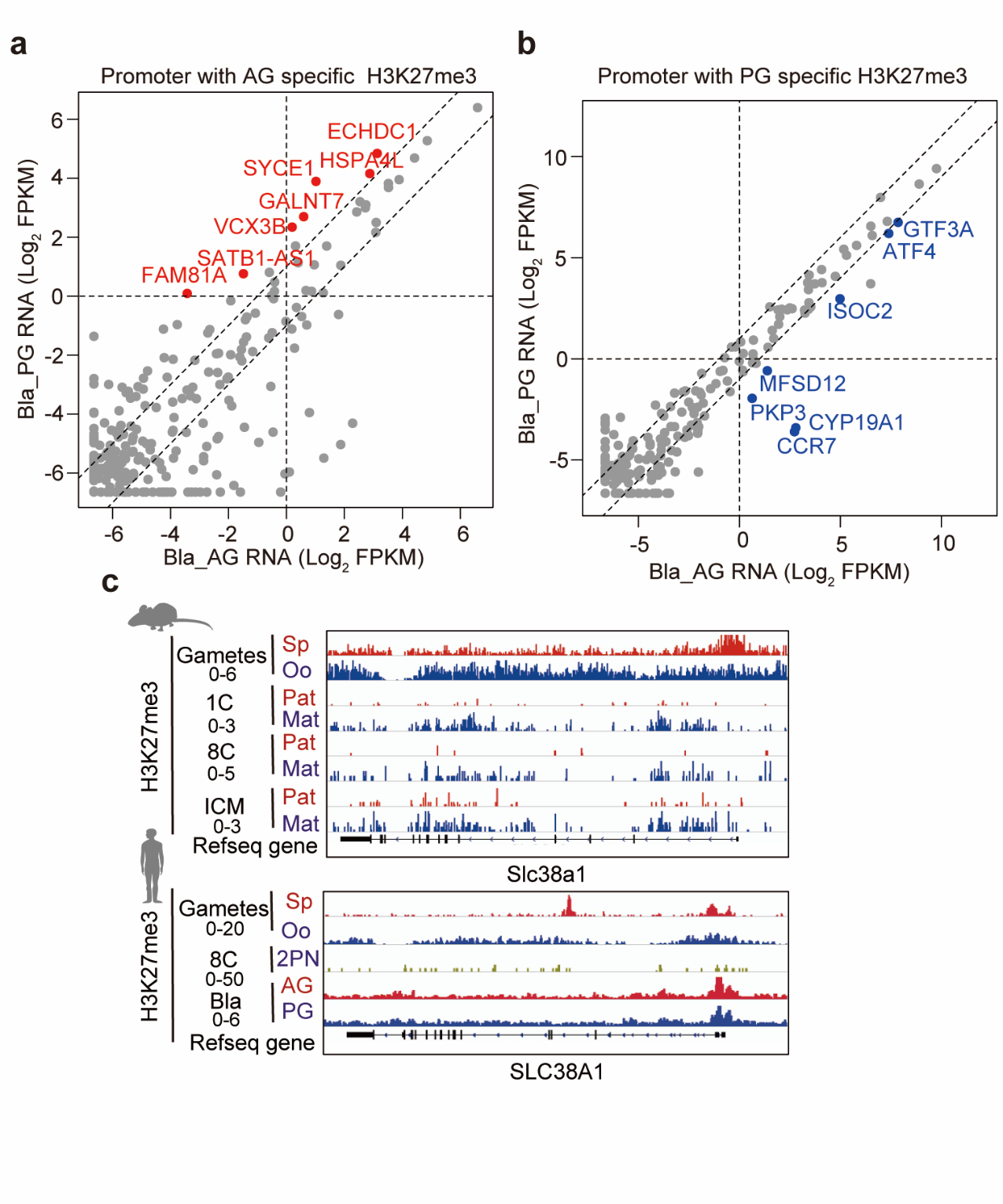
**

**Supplementary Fig. 6 | The parentally specific H3K27me3 signal in human embryos.** **a-b,** The RNA expression levels of the genes with AG-specific promoter H3K27me3 (**a**) or PG-specific promoter H3K27me3 (**b**) in human AG and PG blastocysts. The genes with PG-specific expression are shown in red (**a**). The genes with AG-specific expression are shown in blue (**b**). **c,** Genome browser view of H3K27me3 signal at SLC38A1 loci in mouse and human. SLC38A1 shows H3K27me3-dependent imprinting in mouse.

**Supplementary Reference**

1. Zhong C, Zhang M, Yin Q *et al.* Generation of human haploid embryonic stem cells from parthenogenetic embryos obtained by microsurgical removal of male pronucleus. *Cell Res*. 2016; **26**(6): 743-746. doi: 10.1038/cr.2016.59

2. Zhang XM, Wu K, Zheng Y *et al.* In vitro expansion of human sperm through nuclear transfer. *Cell Res*. 2020; **30**(4): 356-359. doi: 10.1038/s41422-019-0265-1

3. Brind'Amour J, Liu S, Hudson M *et al.* An ultra-low-input native ChIP-seq protocol for genome-wide profiling of rare cell populations. *Nature Communications*. 2015; **6**: 6033. doi: ARTN 6033

10.1038/ncomms7033

4. Liu X, Wang C, Liu W *et al.* Distinct features of H3K4me3 and H3K27me3 chromatin domains in pre-implantation embryos. *Nature*. 2016; **537**(7621): 558-562. doi: 10.1038/nature19362

5. Miura F, Enomoto Y, Dairiki R *et al.* Amplification-free whole-genome bisulfite sequencing by post-bisulfite adaptor tagging. *Nucleic Acids Res*. 2012; **40**(17): e136. doi: 10.1093/nar/gks454

6. Smallwood SA, Lee HJ, Angermueller C *et al.* Single-cell genome-wide bisulfite sequencing for assessing epigenetic heterogeneity. *Nat Methods*. 2014; **11**(8): 817-820. doi: 10.1038/nmeth.3035

7. Yuan S, Zhan J, Zhang J *et al.* Human zygotic genome activation is initiated from paternal genome. *Cell Discov*. 2023; **9**(1): 13. doi: 10.1038/s41421-022-00494-z

8. Gao L, Wu K, Liu Z *et al.* Chromatin Accessibility Landscape in Human Early Embryos and Its Association with Evolution. *Cell*. 2018; **173**(1): 248-259 e215. doi: 10.1016/j.cell.2018.02.028

9. Bolger AM, Lohse M, Usadel B. Trimmomatic: a flexible trimmer for Illumina sequence data. *Bioinformatics*. 2014; **30**(15): 2114-2120. doi: 10.1093/bioinformatics/btu170

10. Langmead B, Salzberg SL. Fast gapped-read alignment with Bowtie 2. *Nat Methods*. 2012; **9**(4): 357-359. doi: 10.1038/nmeth.1923

11. Zhang Y, Liu T, Meyer CA *et al.* Model-based analysis of ChIP-Seq (MACS). *Genome Biol*. 2008; **9**(9): R137. doi: 10.1186/gb-2008-9-9-r137

12. Krueger F, Andrews SR. Bismark: a flexible aligner and methylation caller for Bisulfite-Seq applications. *Bioinformatics*. 2011; **27**(11): 1571-1572. doi: 10.1093/bioinformatics/btr167

13. Hochberg Y, Benjamini Y. More powerful procedures for multiple significance testing. *Stat Med*. 1990; **9**(7): 811-818. doi: 10.1002/sim.4780090710

14. Langmead B, Trapnell C, Pop M *et al.* Ultrafast and memory-efficient alignment of short DNA sequences to the human genome. *Genome Biol*. 2009; **10**(3): R25. doi: 10.1186/gb-2009-10-3-r25

15. John S, Sabo PJ, Thurman RE *et al.* Chromatin accessibility pre-determines glucocorticoid receptor binding patterns. *Nat Genet*. 2011; **43**(3): 264-268. doi: 10.1038/ng.759

16. Ramirez F, Ryan DP, Gruning B *et al.* deepTools2: a next generation web server for deep-sequencing data analysis. *Nucleic Acids Res*. 2016; **44**(W1): W160-165. doi: 10.1093/nar/gkw257

17. Thorvaldsdottir H, Robinson JT, Mesirov JP. Integrative Genomics Viewer (IGV): high-performance genomics data visualization and exploration. *Brief Bioinform*. 2013; **14**(2): 178-192. doi: 10.1093/bib/bbs017

18. Robinson JT, Thorvaldsdottir H, Winckler W *et al.* Integrative genomics viewer. *Nature biotechnology*. 2011; **29**(1): 24-26. doi: 10.1038/nbt.1754

19. Dahl JA, Jung I, Aanes H *et al.* Broad histone H3K4me3 domains in mouse oocytes modulate maternal-to-zygotic transition. *Nature*. 2016; **537**(7621): 548-552. doi: 10.1038/nature19360

20. Tucci V, Isles AR, Kelsey G *et al.* Genomic Imprinting and Physiological Processes in Mammals. *Cell*. 2019; **176**(5): 952-965. doi: 10.1016/j.cell.2019.01.043

21. Hamada H, Okae H, Toh H *et al.* Allele-Specific Methylome and Transcriptome Analysis Reveals Widespread Imprinting in the Human Placenta. *Am J Hum Genet*. 2016; **99**(5): 1045-1058. doi: 10.1016/j.ajhg.2016.08.021

22. Sanchez-Delgado M, Court F, Vidal E *et al.* Human Oocyte-Derived Methylation Differences Persist in the Placenta Revealing Widespread Transient Imprinting. *PLoS Genet*. 2016; **12**(11): e1006427. doi: 10.1371/journal.pgen.1006427

23. Court F, Tayama C, Romanelli V *et al.* Genome-wide parent-of-origin DNA methylation analysis reveals the intricacies of human imprinting and suggests a germline methylation-independent mechanism of establishment. *Genome Res*. 2014; **24**(4): 554-569. doi: 10.1101/gr.164913.113

24. Joshi RS, Garg P, Zaitlen N *et al.* DNA Methylation Profiling of Uniparental Disomy Subjects Provides a Map of Parental Epigenetic Bias in the Human Genome. *Am J Hum Genet*. 2016; **99**(3): 555-566. doi: 10.1016/j.ajhg.2016.06.032

25. Zink F, Magnusdottir DN, Magnusson OT *et al.* Insights into imprinting from parent-of-origin phased methylomes and transcriptomes. *Nat Genet*. 2018; **50**(11): 1542-1552. doi: 10.1038/s41588-018-0232-7

26. Hanna CW, Penaherrera MS, Saadeh H *et al.* Pervasive polymorphic imprinted methylation in the human placenta. *Genome Res*. 2016; **26**(6): 756-767. doi: 10.1101/gr.196139.115
